# Standard rescue medication adjustments produce biased estimates of analgesic efficacy: reducing bias with multiple imputation for rescue adjustment (MIRA)

**DOI:** 10.1097/j.pain.0000000000004074

**Published:** 2026-07-29

**Authors:** Christopher J. Miller, John T. Farrar

**Affiliations:** Department of Biostatistics, Epidemiology, and Informatics, Perelman School of Medicine, University of Pennsylvania, Philadelphia, PA, United States

**Keywords:** Pain measurement, Analgesics, Clinical trials, Acute pain, Postoperative pain, Missing data, Multiple imputation, Reference-based imputation, Rescue medication, Estimands, Intercurrent events, Bias, Research design, Bunionectomy

## Abstract

Acute pain trials rely on imputation procedures to handle the confounding impact of rescue medication on pain scores. Despite long-standing concerns about the bias of single-imputation procedures such as last-, baseline-, and worst-observation carried forward (LOCF, BOCF, WOCF), they remain the standard approaches for rescue adjustment in analgesic trials. To evaluate their performance characteristics, we conducted a data-driven simulation study informed by bunionectomy trials submitted to the U.S. Food and Drug Administration. Our study demonstrates that single-imputation procedures systematically overestimate pain scores after rescue medication use, which produces biased pain curves over time and inaccurate estimates of onset of effect. Because rescue use varies by treatment effectiveness, the direction and magnitude of treatment-effect bias depend on the imputation method. In simulated trials, treatment effects on the sum of pain intensity differences were overestimated by 6% to 7% with LOCF and by 31% to 34% with WOCF and underestimated by 2% to 14% with BOCF depending on the effect size. To address this, we developed Multiple Imputation for Rescue Adjustment (MIRA), a reference-based procedure that uses historical efficacy data on an analgesic to adjust pain scores reported after its use as rescue medication. Multiple Imputation for Rescue Adjustment recovered approximately unbiased estimates under correctly specified simulation conditions and was robust to misspecification of the rescue effect magnitude by up to 50% in either direction. Simulation results were corroborated by reanalyses of 3 independent bunionectomy trials. These findings show that single-imputation methods for handling rescue medication introduce bias in acute pain trials and support MIRA as a more accurate alternative.

## Abstract 2

Supplemental Digital Content is Available in the Text.

Standard approaches to handling rescue medication inflate pain scores and bias treatment effects. A novel reference-based multiple imputation procedure demonstrated superior performance characteristics.

## 1. Introduction

Randomized trials in acute postsurgical pain typically compare an investigational treatment to a placebo or active comparator using an efficacy endpoint based on pain intensity.^2,4,14,20^ Participants repeatedly rate their pain intensity on a numeric rating scale (NRS) or visual analog scale (VAS) over the course of the trial, and these scores are combined into an endpoint such as the sum of pain intensity differences (SPID) or area under the pain intensity curve.

Ethical considerations require that participants not endure unnecessary suffering, so protocols must permit rescue medication when randomized treatment does not provide sufficient pain relief.^15,20^ Because rescue medication affects the pain intensity scores reported, it constitutes an intercurrent event that must be addressed in the analysis. Regulatory agencies recommend prespecification of how intercurrent events such as rescue will be handled to ensure the interpretability of treatment effects.^5,19^

The handling of rescue medication depends on the target estimand.^3,16,19^ The *treatment policy estimand* reflects treatment effects under real-world conditions that incorporate the analgesic effects of all medications, including rescue, so no adjustment for rescue is required. However, participants in less effective treatment groups tend to use rescue more frequently, and this imbalance attenuates between-group differences in pain scores.^15,20,23^ Confirmatory efficacy trials for investigational analgesics in acute pain, therefore, typically target the *hypothetical estimand*, which reflects the intrinsic effect of treatment in a counterfactual scenario without rescue medication.^3,16,19^ Estimating this estimand requires analytic approaches to adjust for the impact of rescue on observed pain scores.

The current standard approaches for handling rescue medication are windowed single-imputation procedures where postrescue pain scores are replaced with the last, baseline, or worst prior pain score reported by the participant (LOCF, BOCF, or WOCF) and carried forward for a prespecified time interval based on the estimated duration of effect of the rescue medication.^15^ Expert panels on missing data have discouraged these methods for imputing missing data in efficacy analyses because of their lack of scientific justification, potential to induce bias, and failure to account for imputation uncertainty.^8,9,13^ Despite these concerns, single-imputation procedures remain the standard practice for handling rescue medication in acute pain trials, and their appropriateness in this setting has not been systematically evaluated.

To address these methodologic issues, we conducted a data-driven simulation study informed by individual participant data (IPD) from bunionectomy trials submitted to the U.S. Food and Drug Administration (FDA). We developed an approach for simulating counterfactual pain scores and realistic rescue-taking behavior to generate trial datasets where the true outcomes for the hypothetical estimand were known. We compared conventional single-imputation procedures with Multiple Imputation for Rescue Adjustment (MIRA), a novel reference-based method that adjusts postrescue pain scores using external data on the analgesic effect of the rescue medication over time. We corroborated simulation findings by applying all methods to empirical data from independent bunionectomy trials. By evaluating key operating characteristics under controlled conditions, we provide evidence to guide method selection in future acute pain trials.

## 2. Methods

### 2.1. Study objectives

The objective of this study was to evaluate the performance characteristics of different methods for handling pain intensity scores after rescue medication use. Efficacy trials for analgesics involve many distinct characteristics such as the surgical context, investigational drugs, choice of rescue medication, inclusion/exclusion criteria, pain assessment schedule, and pain intensity instrument (eg, NRS or VAS). Therefore, we conducted our simulation study under conditions representative of efficacy trials for investigational analgesics, acknowledging that no simulation can cover all possible scenarios.

We selected the bunionectomy surgical model with oral administration of investigational products every 6 hours (q6h) as the trial setting, and hydrocodone/acetaminophen tablets (HC/APAP) as the rescue drug. Bunionectomy is one of the surgical pain models routinely used in confirmatory efficacy trials for new analgesics, where 6-hour dosing of oral analgesics is common.^6,21^ HC/APAP is frequently used as a rescue drug, and an efficacy trial of HC/APAP was available in the FDA dataset for characterizing its analgesic profile. We abstracted summary-level data from this trial using standard statistical procedures, an approach that could be easily applied to determine the analgesic profile of other rescue drugs.

Because the goal of an imputation method for handling rescue is to recover the pain intensity scores that would have been reported if rescue had not been taken, the first stage of the simulation study was to simulate these counterfactual pain scores. The second stage simulated rescue-taking behavior and the expected effect of rescue on pain intensity. We combined both simulated components to produce realistic simulated datasets that mimic the pain scores that would have been observed in actual trials where rescue was used. We then applied each of the methods for handling rescue to the simulated datasets and compared those results with the true counterfactual results to determine performance characteristics. The overall design of the simulation study was informed by best practices outlined by Morris et al.^12^ All code for performing the simulation study is available through GitHub: https://github.com/cjm2718/MIRA.

### 2.2. Data source and population

The simulation study was informed by IPD from previously completed phase 2 and 3 randomized trials of analgesics for the treatment of acute pain that were submitted to the FDA in electronic format as part of new drug applications. As described previously,^11^ deidentified participant-level data from relevant trials were aggregated and harmonized into a master database under contract with the FDA and with the approval of the University of Pennsylvania Institutional Review Board.

The specific bunionectomy trials from the FDA database that were used in this study are described in Table 1. Trials were used for 3 purposes in our study: (1) to estimate the placebo-adjusted effect of the rescue drug (HC/APAP) over time, (2) to estimate the parameters for simulating counterfactual pain intensity scores and rescue-taking behavior, or (3) to independently corroborate the simulation study findings using actual trial data.

**Table 1:** Bunionectomy trial attributes by role in simulation study.

Study ID (Phase)	Study drug; dosing regimen* (n participants)	Rescue medications*	Pain intensity		
			Measure	Enrollment criteria	Scheduled postbaseline assessments
Trial to estimate analgesic effect of hydrocodone/acetaminophen					
CLCT003 (phase 3)	Hydrocodone/acetaminophen/promethazine 7.5/325/12.5; q4-6h over 48 h (252) Hydrocodone/acetaminophen 7.5/325; q4-6h over 48 h (250) Placebo; q4-6h over 48 h (50)	Ibuprofen 400	NRS (0-10)	≥4 on NRS	Every 10 min over the first 2 h, then every 30 min from hours 2 to 6, then every hour while awake through 48 h
Trials with q6h oral dosing to estimate parameters to simulate counterfactual outcomes and rescue-taking behavior					
AP-ADF-105 (phase 3)	Oxycodone/niacin 10/60; q6h over 48 h (135) Oxycodone/niacin 15/60; q6h over 48 h (134) Placebo; q6h over 48 h (136)	IV/IM/PO Ketorolac 10-60	VAS (0-100)	Moderate or severe pain intensity	0.5, 1, 2, 3, 4, 5, 6, and every 6 h through 48 h
ELI-200-003-2014 (phase 3)	Oxycodone/naltrexone 15/1.5; q6h over 48 h (54) Oxycodone/naltrexone 15/3.0; q6h over 48 h (56) Placebo; q6h over 48 h (53)	Ibuprofen 400 IV/IM Ketorolac 15-30	NRS (0-10)	≥4 on NRS	Every half hour through 6 h, 8, 12, 14, 18, 20, 24, 26, 30, 32, 36, 38, 42, 44, and 48 h
ESTEVE-SUSA-301 (phase 3)	Celecoxib/tramadol 112/88; q12h over 48 h (184) Tramadol 50; q6h over 48 h (183) Celecoxib 100; q12h over 48 h (181) Placebo; over 48 h (89)	Acetaminophen 1000 Oxycodone 5	NRS (0-10)	≥5 and ≤9 on NRS	0.25, 0.5, 0.75, 1, 1.25, 1.5, 1.75, 2, 2.5, 3, 3.5, 4, 5, 6, 7, 8, 10, 12, 14, 18, 22, 24, 26, 28, 30, 32, 34, 36, 38, 42, 46, and 48 h
Trials that used hydrocodone/acetaminophen as a rescue drug to corroborate simulation findings					
DIC3-08-04 (phase 3)	Diclofenac 18; q8h over 48 h (109) Diclofenac 35; q8h over 48 h (107) Celecoxib 200; 400 mg loading dose, q12h thereafter (106) Placebo; over 48 h (106)	HC/APAP 10/325 Oxycodone 7.5/325	VAS (0-100)	≥40 on VAS	0.25, 0.5, 0.75, 1, 1.5, 2, 3, 4, 5, 6, 7, 8 12, 16, 20, 24, 32, 40, and 48 h
IND3-08-04b (phase 3)	Indomethacin 20; q8h over 48 h (91) Indomethacin 40; q8h over 48 h (93) Indomethacin 40; q12h over 48 h (91) Celecoxib 200; 400 mg loading dose, q12h thereafter (93) Placebo; over 48 h (94)	HC/APAP 10/325 Oxycodone 7.5/325	VAS (0-100)	≥40 on VAS	0.25, 0.5, 0.75, 1, 1.5, 2, 3, 4, 5, 6, 7, 8, 12, 16, 20, 24, 32, 40, and 48 h
IND3-10-06 (phase 3)	Indomethacin 20; q8h over 48 h (92) Indomethacin 40; q8h over 48 h (94) Indomethacin 40; q12h over 48 h (93) Placebo (94)	HC/APAP 10/325 Oxycodone 7.5/325	VAS (0-100)	≥40 on VAS	0.25, 0.5, 0.75, 1, 1.5, 2, 3, 4, 5, 6, 7, 8, 12, 16, 20, 24, 32, 40, and 48 h

* All doses presented in milligrams (mg).

NRS, numeric rating scale; qXh, every X hours; VAS, visual analog scale.

### 2.3. Overview of simulation approach

Our simulation approach involved 3 sequential stages designed to generate realistic trial data where the true counterfactual outcomes (ie, pain intensity scores without the use of rescue medication) were known. First, we estimated the placebo-adjusted analgesic effect of HC/APAP from historical trial data in which it was administered as the randomized study drug. This placebo-adjusted effect served as the reference distribution for the expected reduction in pain intensity attributable to HC/APAP when used as a rescue drug. Second, we developed a framework to generate realistic counterfactual pain trajectories. These counterfactual trajectories incorporated key features observed in real trials including baseline pain severity, natural pain resolution over time, between-individual heterogeneity in pain trajectories, and within-individual temporal correlation with repeated pain assessments. Third, we simulated rescue-taking behavior based on probabilities of taking rescue medication at different pain intensity levels over time. The rescue medication effects on pain, determined from the first step, were then combined with the counterfactual pain scores to generate simulated data that would be observed in a real trial. This sequential simulation process produced both the true counterfactual pain scores and the observed pain scores reflecting rescue use for each simulated participant. We then applied each method for handling rescue medication to the simulated observed datasets and evaluated how well each recovered the true counterfactual values. Detailed parameter estimation procedures and mathematical specifications are provided in the Supplementary Appendix, http://links.lww.com/PAIN/C546.

### 2.4. Estimating rescue drug effects

We estimated the effect of a single dose of HC/APAP using data from the first dosing interval of CLCT-003, the only bunionectomy trial in the FDA database where HC/APAP was used as the randomized study drug. CLCT-003 was a randomized, double-blind, multiple-dose, placebo- and active-controlled trial among participants experiencing moderate-to-severe pain after bunionectomy. We characterized the analgesic effect using the first dosing interval for 2 reasons. First, all participants were experiencing moderate-to-severe pain at the time they received their first dose, which is consistent with the pain levels at which rescue medication is typically taken. Second, interference from rescue medication was minimized because participants were instructed to avoid taking rescue for at least 90 minutes after the first dose of randomized treatment. Although IPD were available, we used summary-level data so that our approach could be replicated by researchers who have access only to published summaries for other rescue drugs.

We modeled the HC/APAP analgesic effect using both the CLCT-003 data and known pharmacologic properties. We focused on the placebo-adjusted effect, because the analgesic effect of the rescue is in addition to the placebo effect experienced by trial participants. To incorporate the data-driven estimates from CLCT-003 with known pharmacologic information, we used a parametric onset-offset function with a 6-hour duration of action. This function naturally captures the characteristic pharmacodynamic profile of oral analgesics with a gradual onset from baseline, peak effect, and subsequent offset. The model was fit to average pain scores at scheduled time points through 3 hours, after which the analgesic effect was extrapolated to wane through 6 hours. We incorporated variability by applying the standard deviation of pain intensity scores over time from the HC/APAP group to the modeled treatment effect. This approach assumes that participants taking HC/APAP as rescue will experience the placebo-adjusted benefit described by the fitted curve, with individual variation consistent with that observed in the reference trial. Technical details of the model fitting procedure are provided in the Supplementary Appendix, http://links.lww.com/PAIN/C546.

During the development of MIRA, we conducted diagnostic analyses on the CLCT-003 reference data to evaluate the additivity assumption and to assess heterogeneity of the rescue effect across subgroups of interest. We fit subject-level random-intercept generalized additive models to the first dosing interval of CLCT-003, with observations censored after first rescue use. We examined placebo-adjusted treatment-effect curves on the absolute (NRS) scale, overall and stratified by baseline pain intensity (moderate vs severe), age (<40 vs ≥40 years), and sex (female vs male). Treatment-effect curves were similar across baseline-pain strata through the dosing interval. Because a multiplicative effect would be expected to produce larger absolute reductions in the severe baseline pain stratum, the stratified diagnostics supported approximate additivity of the rescue effect. However, we cannot exclude more complex dependence of the rescue effect magnitude on underlying pain intensity. Curves were also similar across age and sex strata, providing no evidence of meaningful heterogeneity by these characteristics. The full model specification, fitted curves, and supporting tables are provided in the Supplementary Appendix, http://links.lww.com/PAIN/C546.

### 2.5. Simulating counterfactual pain scores

Three reference trials using oral q6h dosing of study drugs were available to estimate parameters for simulating counterfactual pain scores and rescue-taking behavior in placebo-controlled bunionectomy trials over 48 hours (Table 1): AP-ADF-105 (oxycodone/niacin), ELI-200-003-2014 (oxycodone/naltrexone), and ESTEVE-SUSA-301 (celecoxib/tramadol cocrystal and individual components). Visual analog scale scores from AP-ADF-105 were converted to NRS equivalents by dividing by 10.

Our objective was to evaluate the performance of imputation methods across a range of potential treatment effects rather than those of specific analgesics. Therefore, we used data from the placebo groups of these reference trials to characterize the dynamics of pain trajectories, including natural pain resolution, between-individual heterogeneity, and within-individual temporal correlation. Parameters based on placebo groups were presumed to most closely reflect the underlying pain process without dependence on active drug effects. We then simulated active treatment groups by applying incremental benefits (small, medium, or large effects) relative to the placebo trajectory using the same structural parameters. This approach allowed us to evaluate the performance of imputation methods across generalized effect sizes. The simulation of counterfactual pain scores incorporated 4 components, as described in the following sections.

#### 2.5.1. Population mean trajectory

The population-level counterfactual mean trajectory for the placebo group followed an exponential decay function reflecting natural recovery from bunionectomy, with mean NRS pain intensity declining from 7 at baseline to 4 at 48 hours. For the first 12 hours, dose-specific effects were imposed to reflect the characteristic pain profile of repeated dosing of oral study medications at 0 and 6 hours.

Active treatment groups were simulated by modifying the 48-hour mean, exponential decay rate, and magnitude of dose effects with the first and second administrations of study drug. Baseline pain and individual-level parameters were held identical to placebo to reflect the expected balance of participant characteristics across randomized groups.

#### 2.5.2. Individual-specific random effects

Heterogeneity between individuals was modeled using individual-specific random effects for baseline pain and slope trajectory. These random effects were drawn from a bivariate normal distribution that was parameterized so that individuals with higher baseline pain tended to experience greater magnitudes of pain reduction, consistent with patterns observed in the 3 reference trials.

#### 2.5.3. Within-individual temporal correlation

Pain scores measured within the same individual are not independent over time. We modeled temporal dependence using a first-order autoregressive [AR(1)] correlation structure, which was supported by autocorrelation patterns observed in the data from the 3 reference trials.

#### 2.5.4. Residual error

After accounting for average population trends, individual-specific trajectories, and temporal correlation, additional within-individual variability was added to reflect the amount of residual variability observed in the reference data.

For each simulated trial participant, pain scores were generated every 15 minutes from baseline through 48 hours. Although pain scores are not collected this frequently in practice, this frequency allowed rescue-taking to be simulated continuously throughout the 48-hour period. For analysis, we extracted pain scores at scheduled assessment timepoints (matched to the assessment schedule from ESTEVE-SUSA-301) and at unscheduled times when rescue was taken for each simulated participant, reflecting the data that would be available in a real trial. The Supplementary Appendix, http://links.lww.com/PAIN/C546, provides details on parameter estimation for counterfactual pain scores from historical trials and the full simulation procedures.

### 2.6. Simulating rescue medication use and rescue medication effects

The probabilities of taking rescue medication were estimated as a function of pain intensity score in the 3 historical bunionectomy trials. For each value of NRS from 0 to 10, we calculated the probability that trial participants would take rescue medication within the next 15 minutes. As expected, the probability of taking rescue was very low at lower pain intensity scores and increased with higher pain scores. No apparent differences in the probability of taking rescue at a particular pain score were observed between randomized treatment groups or at different times. These empirical probabilities were averaged across trials for use in the simulation (Supplemental Table 13, http://links.lww.com/PAIN/C546).

Rescue use for each simulated trial participant was simulated by stepping through the trial one timepoint at a time based on their counterfactual pain scores. After an initial 90-minute rescue lockout period, consistent with usual clinical trial practice, the simulation evaluated each 15-minute timepoint in order. At each timepoint, the probability of rescue medication being taken was determined by the participant's current counterfactual pain score, using the empirical rescue-taking probabilities described above. Once rescue medication was taken, they entered a 6-hour rescue dosing window during which no additional rescue could be taken.

Rescue effects during the 6-hour window were modeled using the HC/APAP reference distribution described previously. These effects were generated using a similar process as described for simulating counterfactual pain scores: the population-level effect over time, between-individual heterogeneity in rescue response (modeled as random intercepts that remained constant across all rescue episodes for each individual), and within-individual temporal correlation with residual variation. The total rescue effect was added to the counterfactual pain scores and rounded to the nearest integer to produce observed pain scores bounded between 0 and 10. This structure allowed us to simulate patients with consistent responses to rescue medication while allowing for realistic variability across different rescue episodes and within each rescue window. Technical details are provided in the Supplementary Appendix, http://links.lww.com/PAIN/C546.

### 2.7. Simulating observed pain scores

Pain scores observed in actual clinical trials reflect the combined effects of the randomized drug and the rescue drug. Therefore, the observed pain scores for each simulated participant were generated by adding the simulated rescue medication effects to the counterfactual pain scores during each rescue window. This additive formulation assumes that the rescue effect operates similarly across randomized treatment groups, with no interaction between rescue medication and study treatment. To create data sets that reflect what would be observed in a real clinical trial setting, simulated pain scores were retained only at scheduled assessment timepoints and at times when rescue was taken. The various methods for handling rescue were then applied to these simulated observed datasets, and the performance of each was evaluated by comparing the pain scores produced by each method with the true counterfactual pain scores.

### 2.8. Simulation design

#### 2.8.1. Primary estimand

The primary estimand was the hypothetical estimand for SPID from 0 to 48 hours (SPID_0-48_), calculated using the standard trapezoidal method. All datasets were generated without intermittent missing data or early withdrawal, so rescue medication was the only intercurrent event that needed to be addressed in the simulations.

#### 2.8.2. Treatment groups and sample sizes

**Figure 1.**
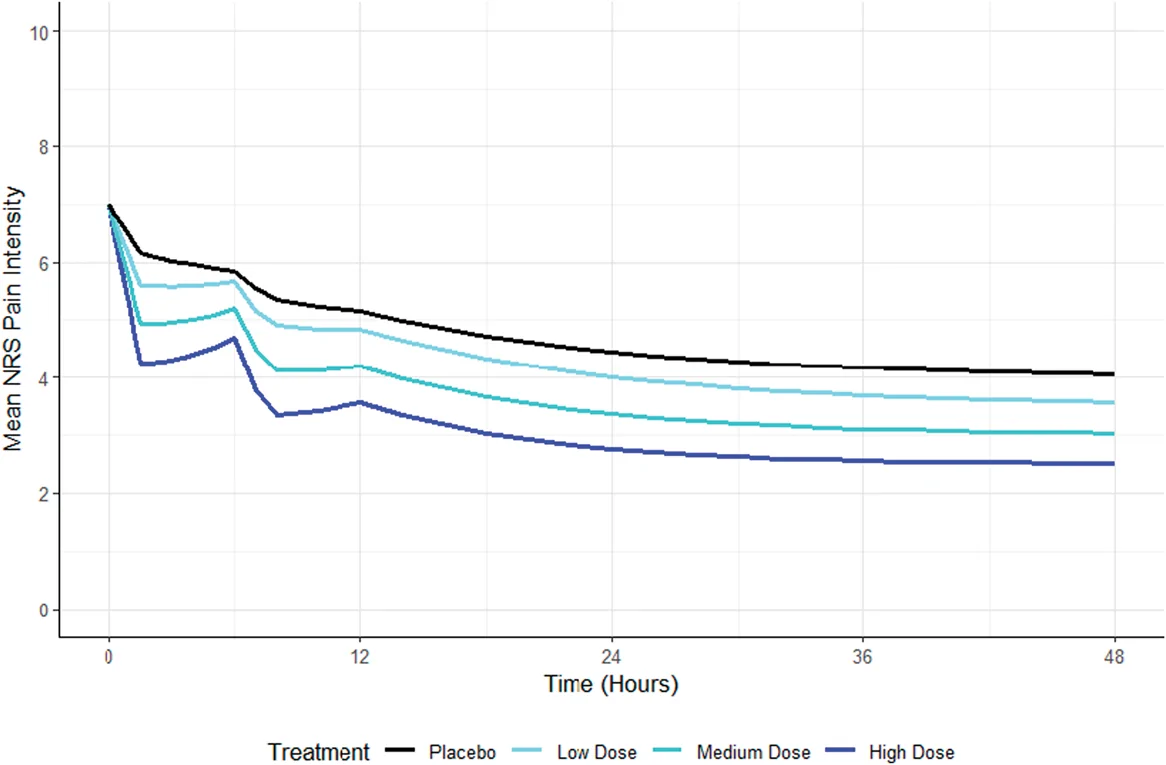
Mean population-level trajectories for simulated treatment groups. NRS, numeric rating scale.

Data were simulated for 4 treatment groups (placebo, low dose, medium dose, and high dose) with placebo-adjusted treatment differences of approximately 0.5, 1.0, and 1.5 NRS points at each timepoint for the low-, medium-, and high-dose groups, respectively. The population mean trajectories for all simulated treatment groups are shown in Figure 1, and the specific parameters used to simulate each of these groups are provided in Supplemental Table 12, http://links.lww.com/PAIN/C546.

Trials were simulated with sample sizes of 50, 100, and 200 per treatment group, which were selected to reflect the range of sample sizes in phase 2 and 3 bunionectomy trials previously submitted to the FDA.

#### 2.8.3. Rescue drug effect magnitude

We evaluated the impact of 3 different magnitudes of the rescue medication effect: (1) as expected, where the population-level effect of rescue was identical to the reference distribution from the CLCT-003 trial; (2) 0.5x expected, where the magnitude of the rescue effect was misspecified to be 50% weaker than expected based on CLCT-003; and (3) 1.5x expected, where the magnitude of the rescue effect was misspecified to be 50% stronger than expected based on CLCT-003. The rationale for these latter 2 scenarios was that MIRA, which uses a reference distribution to adjust for rescue, will either under- or overcorrect for rescue when the magnitude of the reference distribution is misspecified. The peak analgesic effect of HC/APAP in our simulations was −2.2 (NRS scale) for the as-expected case, −1.1 for the 0.5x-expected case, and −3.4 for the 1.5x-expected case. This enabled an evaluation of the robustness of MIRA to misspecifications of the rescue-effect magnitude across a realistic range.

#### 2.8.4. Imputation approaches

Five approaches to handling rescue medication were evaluated by applying each method to every simulated observed dataset and comparing the results to the true counterfactual outcomes.

(1) Observed: no imputation for rescue medication.
(2) LOCF: the last observation at the time rescue medication was taken is carried forward to replace all scheduled pain intensity assessments during each rescue window.
(3) BOCF: the baseline observation is carried forward to replace all scheduled pain intensity assessments during each rescue window.
(4) WOCF: the worst observation (or highest pain score) observed up to the time rescue was taken is carried forward to replace all scheduled pain intensity assessments during each rescue window.
(5) MIRA: the pain scores reported during each rescue window are adjusted using the estimated time-specific efficacy of the rescue drug, derived from the reference distribution.

The single-imputation approaches (LOCF, BOCF, and WOCF) replace observed pain scores after rescue for the duration of the rescue window. Therefore, the underlying assumption of these methods is that the single pain value imputed during the rescue window is the counterfactual pain score that would have been reported had rescue not been taken. Single-imputation approaches further assume no uncertainty in their imputation, because imputed data after rescue are treated identically to observed data unaffected by rescue in the analysis.

Multiple Imputation for Rescue Adjustment is a multiple imputation (MI) approach that adjusts, rather than replaces, observed pain scores within rescue windows. For each scheduled assessment within a rescue window, MIRA draws a time-specific rescue effect estimate from the reference distribution (see Section 2.4) based on the time since taking rescue. Rescue medication reduces pain, so the rescue effect will generally be negative. Subtracting this negative effect from the observed pain score adds back the expected analgesic benefit, recovering the estimated counterfactual pain score. Pain intensity assessments outside rescue windows are unchanged. As an MI procedure, this process is repeated to create multiply imputed datasets, each of which is analyzed identically, with results combined using standard MI rules. Thus, MIRA adds back the expected time-specific rescue effects to observed postrescue pain scores to recover the counterfactual values, accounting for imputation uncertainty. In this simulation study, MIRA was performed using 100 imputed datasets for each simulated trial.

#### 2.8.5. Statistical analysis

For each simulated trial and imputation method, SPID_0-48_ values were analyzed using an ANCOVA model with treatment group as a main effect controlling for baseline pain score. For MIRA, ANCOVA estimates across the imputed datasets were combined using Rubin's rules.^10^

#### 2.8.6. Simulation sample size

For each combination of conditions (sample size per group, treatment effect size, and rescue effect size), individual participant data were generated for 5000 simulated trials. This simulation sample size was selected to ensure that Monte Carlo uncertainty would be much smaller than a practically important difference between different methods for handling rescue.^12^

#### 2.8.7. Performance characteristics

We evaluated the performance of the various imputation approaches to understand their ability to estimate both treatment group-level estimates and placebo-adjusted treatment differences.

Bias was calculated as the difference between the true counterfactual SPID value and the imputed SPID value in each simulated trial. Because the absolute bias increases as a function of the treatment effect, we also calculated the relative (percent) bias. We calculated empirical standard errors, root mean square error (RMSE), and coverage probabilities of 95% confidence intervals (CIs).

Type I error was estimated using a null treatment group with the same data-generating parameters as the placebo group. The type I error rate was defined as the proportion of trials where a statistically significant result was observed in the absence of a true effect. Statistical power was estimated as the proportion of trials where a statistically significant result was observed when a true effect existed.

Finally, because the SPID metric is rarely viewed graphically, we sought to illustrate the impact of the different imputation approaches on pain curves over time. To accomplish this, we simulated 2000 participants per treatment group for each rescue effect level to reduce the sampling variability and show the average pain trajectories across the methods.

### 2.9. Corroborating simulation results with independent trials

Simulation studies rely on simplifying clinical reality in ways that can be evaluated under controlled statistical settings where the counterfactual truth is known. Complementing simulations with real-world data applications is critical to assess whether simulation findings hold under the full complexity of actual trial data. To corroborate our simulations, we applied all imputation approaches to 3 trials that used HC/APAP as the primary rescue medication that were independent of the simulation study (Table 1). In these trials, a higher dose of HC/APAP was used as rescue (10/325 mg vs 7.5/325 mg in the reference trial) and oxycodone/APAP (10/325 mg) was available as a secondary rescue medication. Thus, the reference rescue distribution for MIRA may underestimate the true rescue effect but is likely within the 50% magnitude range for misspecification that was evaluated in the simulation study. Unlike the simulated data, some participants dropped out of the study early. Therefore, for all methods for rescue (observed, single-imputation methods, and MIRA), missing data because of dropout were handled using multiple imputation by predictive mean matching, with baseline pain intensity, time of dropout, and pain intensity at time of dropout as predictors in the imputation model.

## 3. Results

For each section below, we first present simulation results for treatment effects on SPID_0-48_ (ie, mean differences between the active and placebo groups) and then examine treatment-group–level estimates. These 2 quantities are subject to different patterns of imputation bias, where the bias in treatment effects depends on differences in rescue use between groups, and bias in treatment-group estimates reflects the extent of rescue use within each group.

### 3.1. Summary results with sum of pain intensity differences

**Figure 2.**
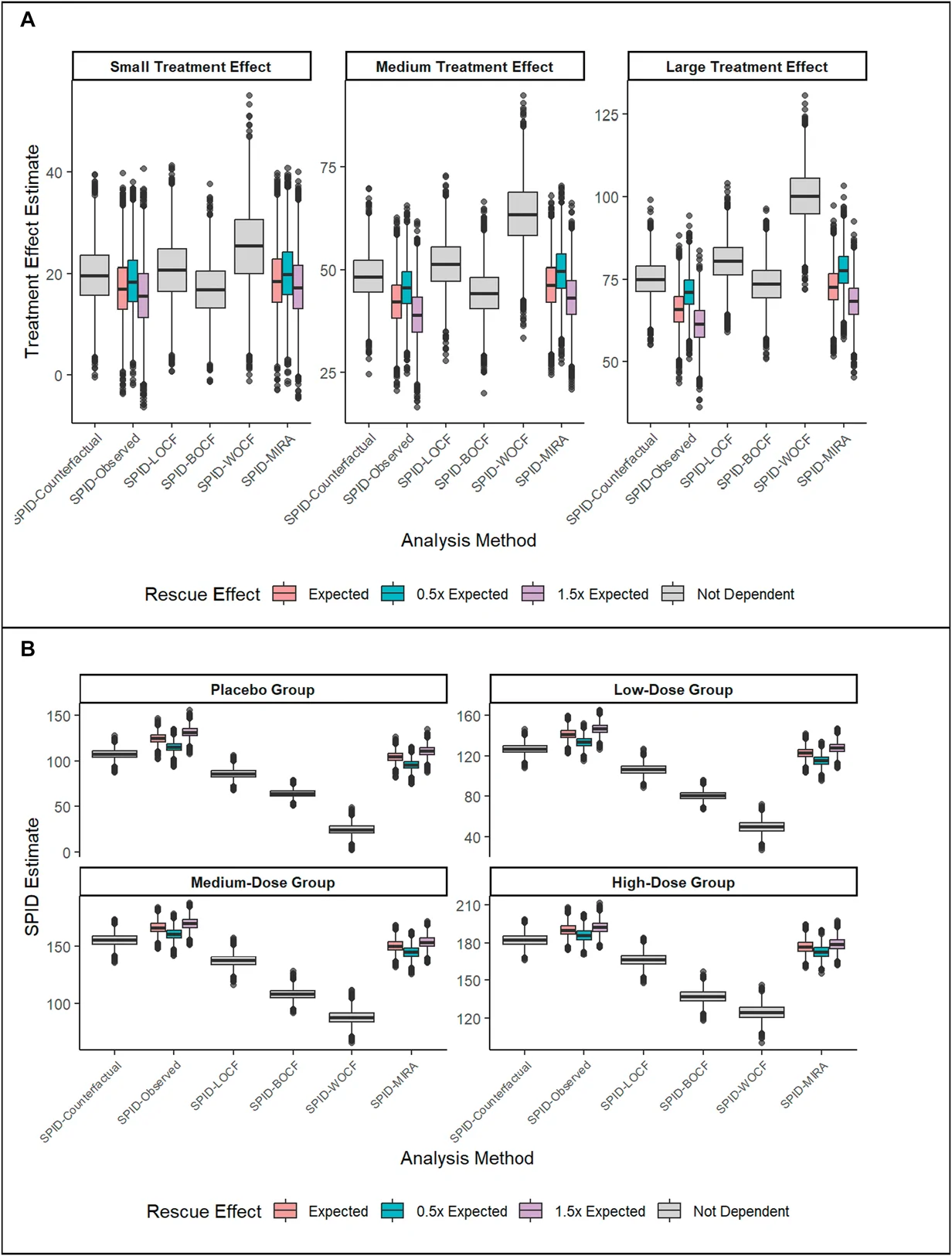
Box plots of SPID for treatment contrasts vs placebo (Panel A) and group-level estimates (Panel B). Data represent simulated trials with N = 200 per treatment group. SPID, sum of pain intensity differences.

Averaged across simulated trials, the true counterfactual (no-rescue) treatment effect on SPID_0-48_ was approximately 19 for the small effect (low dose vs placebo; average difference of 0.5 NRS), 48 for the medium effect (medium dose vs placebo; average difference of 1.0 NRS), and 75 for the large effect (high dose vs placebo; average difference of 1.5 NRS) (Fig. 2A). The SPID treatment-effect estimates based on observed data without imputation or BOCF for rescue tended to underestimate the counterfactual effect, whereas estimates based on LOCF and WOCF tended to overestimate it. Estimates based on MIRA closely matched the counterfactual effect, with only modest deviations even under 50% misspecification of the rescue-effect magnitude in either direction.

Averaged across simulated trials, the true counterfactual SPID_0-48_ values were 107 for the placebo group, 127 for the low-dose group, 156 for the medium-dose group, and 182 for the high-dose group (Fig. 2B). The SPID values for each treatment group based on observed data without imputation were overestimated relative to the true counterfactual because the pain-reducing effects of rescue were not accounted for. Treatment group SPID values based on windowed LOCF, BOCF, and WOCF for handling rescue all underestimated the true counterfactual values. SPID values based on MIRA closely matched the true counterfactual group values, with little impact from misspecification of the rescue effect magnitude.

### 3.2. Bias

**Figure 3.**
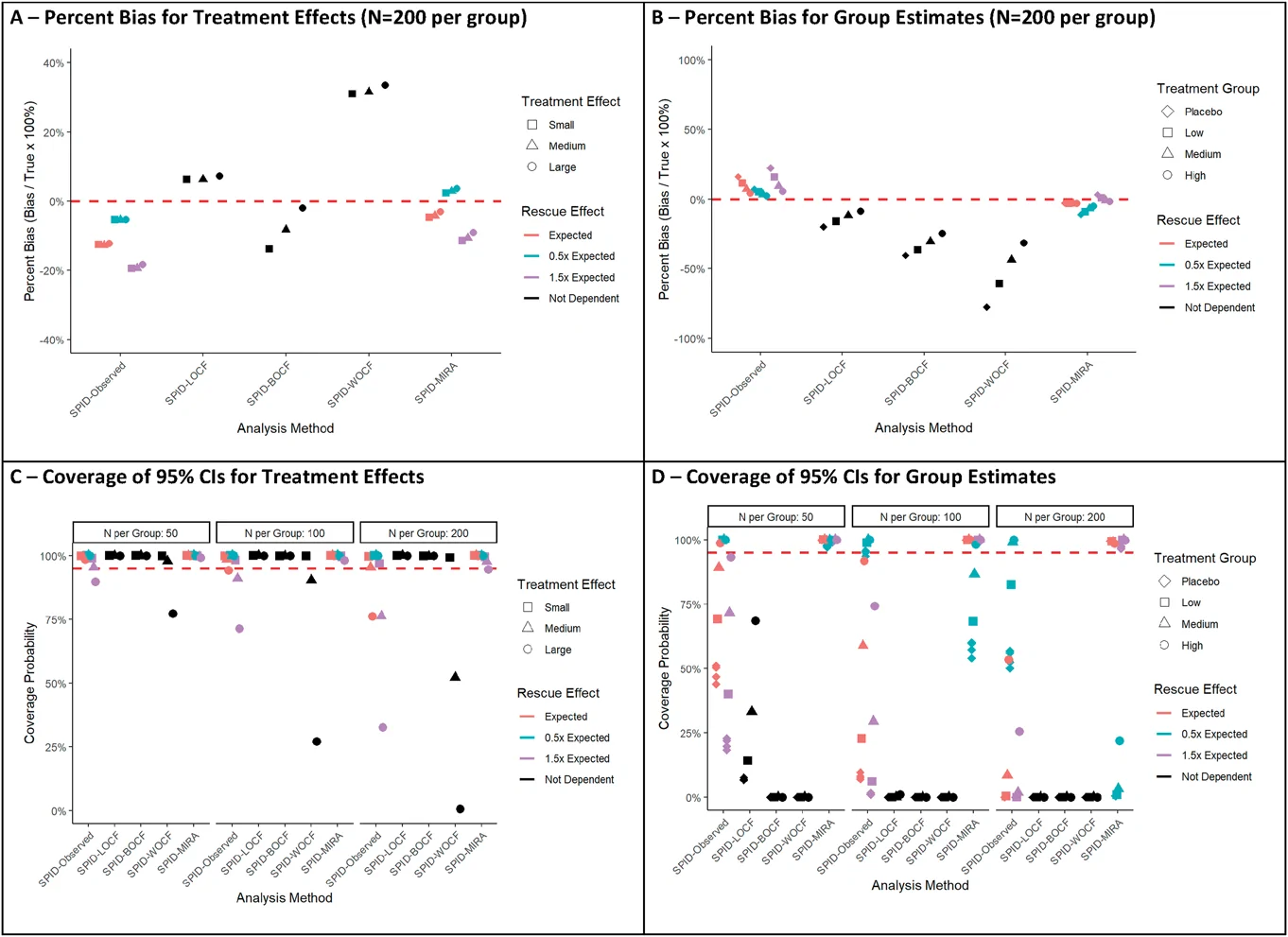
Percent bias for treatment effects (Panel A), percent bias for treatment group-level estimates (Panel B), coverage of 95% CIs for treatment effects (Panel C), and coverage of 95% CIs for treatment group-level estimates (Panel D). Because percent bias is not affected by sample size, only results for trials with N = 200 per group are shown for Panels (A and B).

The patterns of bias for treatment effect estimates on SPID varied as a function of the difference in the amount of rescue use between groups (Fig. 3A). The use of observed data without imputation consistently underestimated the true counterfactual treatment effect, with larger rescue effects producing greater bias. Among the windowed single-imputation methods, the direction of the bias depended on which pain value was carried forward. The SPID treatment effects based on LOCF and WOCF overestimated the treatment effect, by 6% to 7% with LOCF and by 31% to 34% with WOCF. The BOCF method showed conservative bias, underestimating treatment effects by 2% to 14%. The SPID treatment effects based on MIRA had minimal bias (3%-5% conservative) when correctly specified and showed 9% to 11% conservative bias when the true rescue effect exceeded the reference distribution (1.5× expected).

The bias in treatment group estimates for SPID varied as a function of the amount of rescue taken within each group (Fig. 3B). Analyses of observed data without imputation overestimated SPID in all groups, with placebo groups showing the largest overestimation because of their higher use of rescue. The 3 single-imputation methods underestimated the true SPID values in all treatment groups. The range of relative bias across the different treatment groups was −9% to −20% for LOCF, −25% to −41% for BOCF, and −32% to −78% for WOCF. Across all single-imputation methods, bias was consistently most severe in the placebo group, reflecting higher rescue medication use in the placebo group than in active drug groups. Treatment group values for SPID estimated using MIRA achieved minimal bias (within ±3%) across all treatment groups when the rescue effect was correctly specified. When rescue effects were underestimated by half (0.5× expected), MIRA showed modest conservative bias reaching −11% in the placebo group but was more accurate than any single-imputation method.

### 3.3. Coverage rate for 95% confidence intervals

For SPID treatment differences vs placebo, single-imputation methods achieved near-nominal or conservative (>95%) coverage (Fig. 3C), as much of the bias introduced by the different methods for rescue adjustment cancels out in between-group differences. The MIRA method maintained approximately nominal 95% coverage in all scenarios, regardless of correct specification or 50% over- or underspecification of the rescue effect distribution.

The coverage probabilities for treatment-group–level SPID estimates were consistent with the findings for bias. The SPID estimates for treatment groups based on single-imputation methods for rescue medication showed very poor coverage (Fig. 3D), with 0% coverage in most scenarios. In contrast, MIRA achieved nominal 95% coverage when rescue effects were correctly specified or 1.5× expected but showed some undercoverage at larger sample sizes when the rescue effect was 0.5× expected.

### 3.4. Empirical standard errors

As anticipated, the standard errors decreased for all methods as a function of the treatment group sample size (Supplemental Fig. 14, http://links.lww.com/PAIN/C546). Empirical standard errors were generally comparable across the methods of adjusting for rescue except for WOCF, which showed elevated standard errors because of the imputation of extreme values. The MIRA method did not show substantial variance inflation despite accounting for uncertainty with multiple imputation, yielding standard errors similar to the single-imputation methods while achieving superior bias properties. The findings with RMSE were congruent with those for bias and empirical standard errors (Supplemental Fig. 15, http://links.lww.com/PAIN/C546).

### 3.5. Type I error and power

All methods for handling rescue maintained adequate type I error control, with 95% Monte Carlo CIs for the type I error rate at or below the nominal 5% level (Supplemental Fig. 16A, http://links.lww.com/PAIN/C546). The MIRA method showed slightly conservative type I error rates (approximately 4.0%-4.5%), a known phenomenon when using Rubin's rules with reference-based multiple imputation.^1^

As expected, statistical power varied primarily with the treatment effect size and sample size. No method for handling rescue medication showed a clear advantage for power (Supplemental Fig. 16B, http://links.lww.com/PAIN/C546).

### 3.6. Illustration of simulation results with pain curves over time

**Figure 4.**
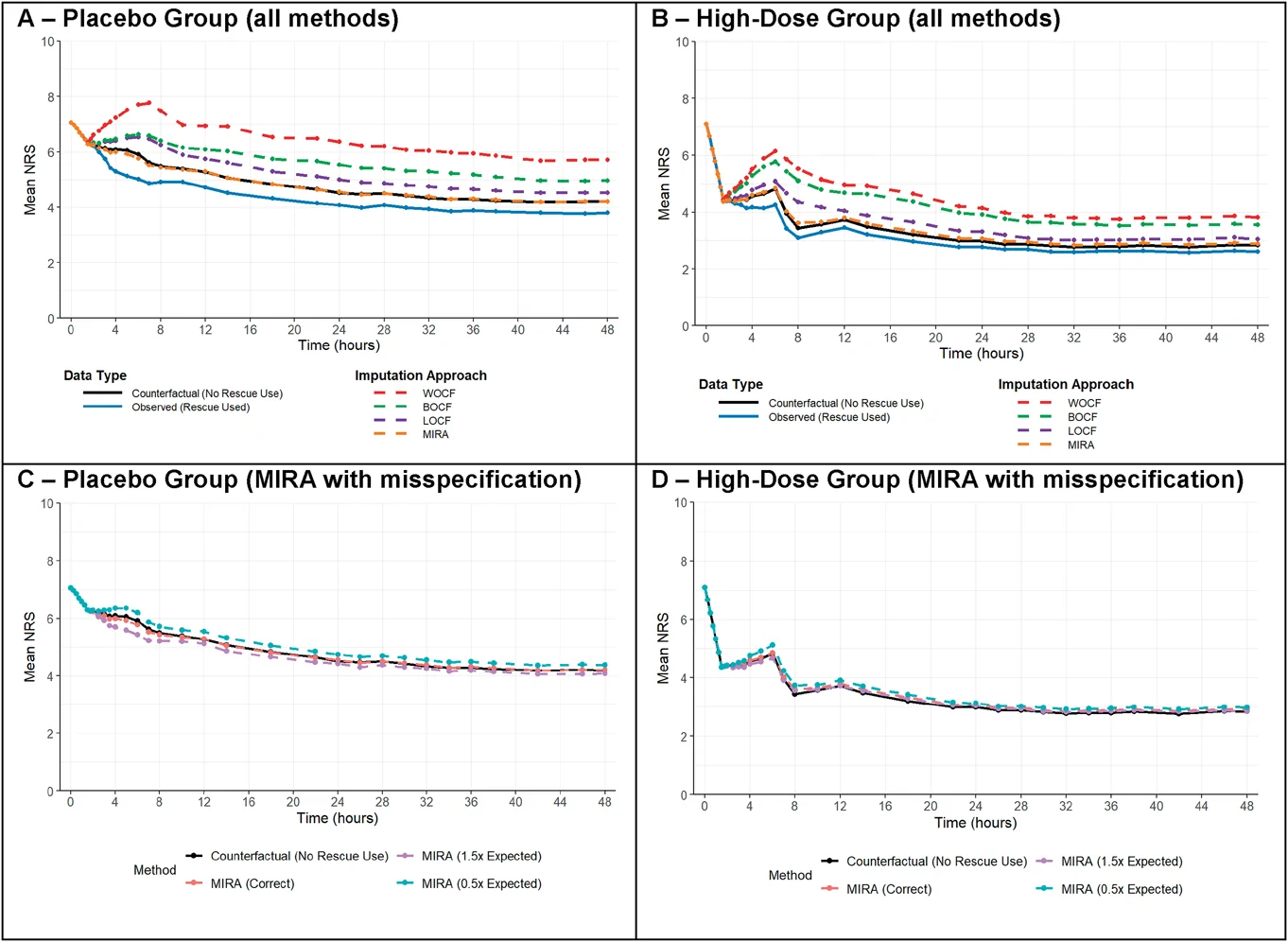
Mean pain scores over time by data type and imputation method for the simulated placebo group (Panel A), the simulated high-dose group (Panel B), MIRA for the simulated placebo group under correct specification and misspecification (Panel C), and MIRA for the simulated high-dose group under correct specification and misspecification (Panel D). MIRA, multiple imputation for rescue adjustment.

To visualize the impact of bias for the various methods of handling rescue, we generated pain intensity curves over time with 2000 simulated participants per group (to minimize sampling variability and isolate the effects of bias) for placebo (Fig. 4A) and high-dose conditions (Fig. 4B). Consistent with the results for bias, the analysis of the observed data without adjustment for rescue medication (blue line) overestimated treatment benefit. The WOCF method for handling rescue (dashed red line), which also had the greatest bias for estimating group-level SPID estimates, had the largest deviation from the true counterfactual pain scores, followed by BOCF (dashed green line) and LOCF (dashed purple line). The mean pain curve based on MIRA imputation for rescue (dashed orange line) closely tracked the counterfactual trajectory in both treatment groups, confirming the unbiased estimation when rescue effects were correctly specified.

Panels C and D of Figure 4 examine the robustness of MIRA to misspecification of the true rescue effect distribution for the placebo and high-dose groups, respectively. Misspecification of the magnitude of the rescue effect by 50% in either direction had relatively modest effects on the estimated mean pain scores.

### 3.7. Imputation applications to independent trials

**Figure 5.**
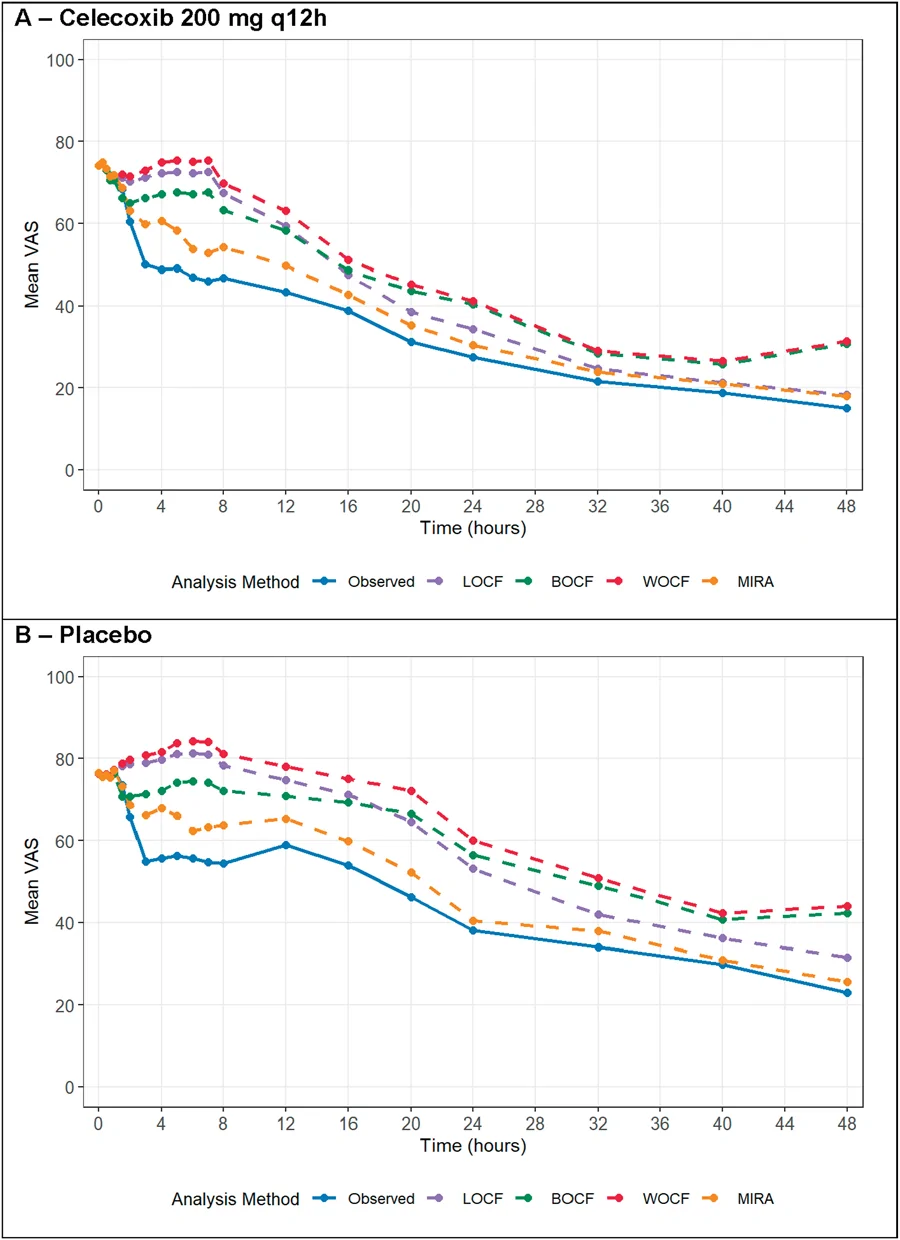
Mean VAS pain scores over time by analysis method for the celecoxib 200 mg q12h group (Panel A) and the placebo group (Panel B) of study DIC3-08-04. VAS, visual analog scale.

To evaluate the impacts of the different rescue imputation methods on real data, we analyzed 3 independent bunionectomy trials for which HC/APAP was the rescue medication. The results from these reanalyses were consistent with the simulation results for SPID and pain intensity curves. As shown by representative pain curves for the celecoxib 200 mg q12h and placebo groups from DIC3-08-04 (Fig. 5), pain scores based on observed data without imputation for rescue were lowest, followed by MIRA (which was unbiased in the simulations) and then the single-imputation methods (which substantially overestimated average pain intensity scores for treatment groups in the simulations). Results based on windowed LOCF, BOCF, and WOCF methods for handling rescue would lead to a conclusion that celecoxib had minimal benefit until 8 hours post dosing, but results based on MIRA suggest that mean pain scores started to gradually decline immediately after dosing. Although counterfactual results cannot be known with real-world data, the estimates reflected by MIRA are more clinically and pharmacologically plausible than the conclusion that celecoxib had an 8-hour delay of onset when used in bunionectomy.

By 2 hours after baseline in study DIC3-08-04, approximately 60% of participants in the celecoxib group and 80% in the placebo group had taken rescue medication (Supplemental Fig. 17, http://links.lww.com/PAIN/C546). The apparent improvements in pain scores at 8 hours with the single-imputation methods for rescue, therefore, reflect a statistical artifact of imputing high pain scores that remain fixed for 6 hours after the first use of rescue. Although MIRA uses the same 6-hour rescue window, the application of the reference HC/APAP rescue effect yielded estimates that align with clinical and pharmacological expectations. Similar findings were observed for other treatment groups in DIC3-08-04 (Supplemental Fig. 18, http://links.lww.com/PAIN/C546) and in the other 2 trials, IND3-08-04b (Supplemental Fig. 19, http://links.lww.com/PAIN/C546) and IND3-10-06 (Supplemental Fig. 20, http://links.lww.com/PAIN/C546).

For SPID_0-48_, the estimated treatment effects (Supplemental Fig. 21, http://links.lww.com/PAIN/C546) and group-level estimates (Supplemental Fig. 22, http://links.lww.com/PAIN/C546) from the real-world trials aligned with the simulation study results. The MIRA method consistently fell between the estimates for observed data without rescue imputation and the 3 windowed single-imputation methods.

## 4. Discussion

This data-driven simulation study showed that the windowed single-imputation procedures commonly used to adjust for rescue medication in acute pain trials induce substantial bias in both treatment-group outcomes and treatment effect estimates. These imputation methods systematically overestimate pain scores during postrescue periods. Because the magnitude of pain score inflation corresponds to the extent of rescue use, the bias induced by imputation is differential by treatment group, leading to distorted treatment effect estimates. In addition, because rescue utilization tends to be high early in the study, these biases produce incorrect estimates of analgesic onset in pain intensity curves. The LOCF and WOCF procedures led to overestimation of the true placebo-adjusted treatment effect by 6% to 7% and 31% to 34%, respectively, whereas BOCF underestimated treatment effects by 2% to 14%. In contrast, MIRA produced unbiased estimates for treatment-group outcomes and treatment effects when the rescue effect distribution was correctly specified and demonstrated robust inference even under misspecification of the magnitude of the rescue effect by 50% in either direction. Simulation findings were also corroborated by reanalyses of 3 independent bunionectomy trials.

Windowed single-imputation procedures for handling missing data in clinical trials are widely recognized as biased and discouraged by regulatory agencies and statistical experts.^7–10^ Although contemporary trials generally use more principled statistical approaches for addressing missing data, these same single-imputation procedures remain the standard methods for handling rescue medication in acute pain trials to estimate the pure drug effect on pain (ie, the hypothetical estimand). Despite their known limitations, no suitable alternatives to single-imputation procedures have been available to address rescue. These procedures are consistent with the perspective that taking rescue medication represents a treatment failure and warrants a punitive adjustment. However, applying excessive penalties for rescue use creates biased estimates for treatment effects because the bias introduced by single-imputation procedures is smallest for the most effective treatments and greatest for placebo. As a result, placebo-adjusted treatment effects, which form the primary basis for regulatory decision making in acute pain trials, are particularly sensitive to imputation bias, with the magnitude of bias proportional to differences in rescue use between groups.

The MIRA method is a novel reference-based multiple imputation approach that offers several methodological advantages. First, it explicitly acknowledges uncertainty through the multiple imputation framework, properly accounting for imputation uncertainty in standard errors and confidence intervals. Second, MIRA leverages the observed postrescue pain scores to estimate the counterfactual. For example, consider 2 participants with equivalent baseline pain who both take rescue at the same time and pain level, where one participant had received an active drug with a 60-minute onset and the other had received placebo. The active participant will likely have a steeper decline in pain after rescue as their drug takes effect along with the rescue drug. The MIRA method preserves this distinction, whereas any single-imputation procedure would replace postrescue scores during the rescue window with the same value for both participants. Our study also suggests that adjusting observed postrescue scores with MIRA rather than replacing them prevents the inflation of standard errors that can occur with reference-based imputation. Finally, MIRA's assumptions about rescue drug effects better reflect clinical and pharmacological reality, where analgesics have characteristic onset, peak, and offset patterns. This represents a fundamental improvement over single-imputation procedures, which implausibly assume that pain levels would have remained constant for a set time period after each rescue dose.

These findings have important implications for the interpretation of acute pain trial results. Although SPID is typically the primary endpoint used for statistical comparisons, SPID values themselves have limited clinical interpretability.^22^ As a result, investigators and clinicians rely on pain intensity curves over time to assess clinical importance of pain scores and relative treatment benefits. However, published pain curves typically apply single-imputation procedures to handle rescue, producing results that our study demonstrates are systematically biased. This was illustrated empirically in the reanalyses of the bunionectomy trials, where substantial early rescue use caused single-imputation methods to produce distorted pain curves that falsely indicated analgesics had a delayed onset of action and offered no benefit during the first several hours after surgery. In contrast, pain curves based on MIRA reflected a modest benefit after dosing that increased over time, consistent with the known pharmacologic properties of the randomized drugs. Readers should be aware that pain intensity curves generated with single-imputation procedures to handle rescue medication systematically overstate pain levels in all treatment groups. Because the magnitude of bias from single-imputation procedures is proportional to the extent of rescue use, the degree of distortion will vary across trials and treatment comparisons. This has important implications for meta-analyses, cross-trial comparisons, and comparative effectiveness research, because variable treatment effects reported across studies could be influenced by differences in rescue utilization patterns or imputation practices rather than true differences in analgesic efficacy.

### 4.1. Limitations and future directions

Counterfactual pain outcomes in the absence of rescue medication are unknowable in real-world data because of the ethical requirements of clinical research, so simulation studies must rely on simplifying assumptions. A strength of the present study is that simulation parameters were informed by IPD from 3 registration trials submitted to the FDA. The rescue effect distribution for HC/APAP, however, was derived primarily from summary statistics from a single trial that was itself subject to the confounding effects of rescue medication use. To partially address this, we restricted estimation of the rescue effect to timepoints through 3 hours and used the upper limit of the dosing interval to inform the offset. The MIRA method demonstrated robust performance under misspecification of the rescue effect magnitude by up to 50% in either direction, with peak average effects ranging from 1.1 to 3.4 points on the NRS, covering the plausible range for a single oral dose of most analgesics. Nonetheless, sensitivity analyses around the rescue effect distribution should be performed in analyses of future trials to confirm the robustness of results.

Our sensitivity analyses for misspecification in this paper introducing the MIRA approach were restricted to evaluation of scalar shifts in the magnitude of the rescue effect distribution and did not evaluate alternative functional forms. This approach introduces partial alignment between the data-generating mechanism and the imputation model, which inherently favors MIRA. Although this assumption may be reasonable given that the pharmacodynamic effects of oral analgesics are well known, the true effect of an analgesic used as rescue medication could deviate from the pharmacodynamic profile established in placebo-controlled trials because of contextual factors of rescue use (eg, expectancy effects with open-label treatment, affective aspects of pain that interact with rescue-taking). Similarly, our simulation framework and MIRA both assume that the rescue effect operates similarly in participants randomized to active treatment and placebo. Rescue pain medications are typically chosen from a different drug class than the investigational agent and should be selected to minimize the potential for pharmacologic interaction. Although our analyses showed that the probability of taking rescue at a given pain level was similar for active drug and placebo groups, it is possible that the rescue effect itself is differential. Evaluating MIRA under more diverse functional misspecifications and under richer sources of uncertainty is an area for future methodological work.

Another limitation is that the MIRA framework as implemented here is not directly applicable to trials of chronic pain. Chronic pain trials differ fundamentally in their durations, patterns of rescue medication use, and assessment of pain intensity (eg, frequent assessments of current pain throughout the day in acute pain trials vs typically daily assessments of average pain in chronic pain trials). Recent work has begun to address the challenges of handling rescue medication in chronic pain trials,^17,18^ and this remains an important area for future methodological development.

Several extensions of this work would strengthen the evidence base for and utility of MIRA. Systematic literature reviews could characterize effects of other common rescue medications (eg, ibuprofen, oxycodone) and define standard rescue effect distributions for future trials. Additional simulations could explore a broader set of parameters and applications of MIRA beyond bunionectomy. Although confidentiality restrictions preclude us from making the IPD from these reference trials publicly available, the Supplementary Appendix, http://links.lww.com/PAIN/C546, provides extensive details to inform additional work. Importantly, MIRA requires no specialized software and can be implemented using standard multiple imputation functions available in any commercial statistical software. We have made our simulation code in R publicly available to support continued development and to facilitate implementation.

### 4.2. Conclusion

This simulation study found that single-imputation methods, the current standard approaches for handling rescue medication in acute pain trials, introduce substantial bias in both treatment group and treatment effect estimates. We introduced MIRA, a reference-based multiple imputation approach that provided unbiased estimates and was robust to misspecification of the rescue effect magnitude. Simulation results were corroborated by reanalyses of individual participant data from 3 independent bunionectomy trials. Together, these findings support the use of MIRA as a principled alternative to single-imputation procedures for handling rescue medication in acute pain trials.

## Conflict of interest statement

C.J.M. was previously employed by Pacira BioSciences. The work reported in this manuscript was completed before his employment at Pacira, which had no role in the study design, analysis, interpretation of results, or decision to submit the manuscript for publication. J.T.F. reports that over the past 3 years, he has received funding from NIH-NCATS—UL1 Grant (Co-I), NIH-NIDDK—U01 Grant (Co-I), NIH-NINDS—U24 Grant (PI), and 2 FDA-BAA Contracts, and compensation for serving on advisory boards or consulting on clinical trial methods from Vertex, EicOsis, 3Daughters, Scilex Holding Company, and Lilly. He is the past President of the United States Association for the Study of Pain.

## Supplementary Material

## Appendix A. Supplemental digital content

Supplemental digital content associated with this article can be found online at http://links.lww.com/PAIN/C546.

## References

[1] Bartlett JW. Reference-based multiple imputation—what is the right variance and how to estimate it. Stat Biopharm Res 2023;15:178–86.

[2] Cooper SA Desjardins PJ Turk DC Dworkin RH Katz NP Kehlet H Ballantyne JC Burke LB Carragee E Cowan P Croll S Dionne RA Farrar JT Gilron I Gordon DB Iyengar S Jay GW Kalso EA Kerns RD McDermott MP Raja SN Rappaport BA Rauschkolb C Royal MA Segerdahl M Stauffer JW Todd KH Vanhove GF Wallace MS West C White RE Wu C. Research design considerations for single-dose analgesic clinical trials in acute pain: IMMPACT recommendations. PAIN 2016;157:288–301. 26683233 10.1097/j.pain.0000000000000375

[3] European Medicines Agency. ICH E9 (R1) addendum on estimands and sensitivity analysis in clinical trials to the guideline on statistical principles for clinical trials. European Medicines Agency; https://www.ema.europa.eu/en/documents/scientific-guideline/ich-e9-r1-addendum-estimands-and-sensitivity-analysis-clinical-trials-guideline-statistical-principles-clinical-trials-step-5_en.pdf (2020, Accessed July 8, 2026).

[4] Gilron I Carr DB Desjardins PJ Kehlet H. Current methods and challenges for acute pain clinical trials. PAIN Rep 2019;4:e647. 31583333 10.1097/PR9.0000000000000647 PMC6749920

[5] International Council for Harmonisation of Technical Requirements for Pharmaceuticals for Human Use. ICH E9 (R1) addendum on estimands and sensitivity analysis in clinical trials to the guideline on statistical principles for clinical trials. Available at: https://database.ich.org/sites/default/files/E9-R1_Step4_Guideline_2019_1203.pdf. Accessed November 12, 2025.

[6] Jones J Correll DJ Lechner SM Jazic I Miao X Shaw D Simard C Osteen JD Hare B Beaton A Bertoch T Buvanendran A Habib AS Pizzi LJ Pollak RA Weiner SG Bozic C Negulescu P White PF. Selective inhibition of Na(V)1.8 with VX-548 for acute pain. N Engl J Med 2023;389:393–405. 37530822 10.1056/NEJMoa2209870

[7] Lachin JM. Fallacies of last observation carried forward analyses. Clin Trials 2016;13:161–8. 26400875 10.1177/1740774515602688 PMC4785044

[8] Little RJ Cohen ML Dickersin K Emerson SS Farrar JT Neaton JD Shih W Siegel JP Stern H. The design and conduct of clinical trials to limit missing data. Stat Med 2012;31:3433–43. 22829439 10.1002/sim.5519 PMC5944851

[9] Little RJ D'Agostino R Cohen ML Dickersin K Emerson SS Farrar JT Frangakis C Hogan JW Molenberghs G Murphy SA Neaton JD Rotnitzky A Scharfstein D Shih WJ Siegel JP Stern H. The prevention and treatment of missing data in clinical trials. N Engl J Med 2012;367:1355–60. 23034025 10.1056/NEJMsr1203730 PMC3771340

[10] Little RJA Rubin DB. Statistical analysis with missing data. Hoboken, NJ: Wiley, 2020.

[11] Miller CJ Bilker WB DeLorey I Argoff CE Bell RL Conroy A Gewandter JS Gilron I Haythornthwaite JA Katz NP McWilliams T Theken KN Farrar JT. Minimum clinically important differences in acute pain: a patient-level re-analysis of randomized controlled analgesic trials submitted to the US Food and Drug Administration. PAIN 2025;166:2430–8. 40359379 10.1097/j.pain.0000000000003645 PMC12228562

[12] Morris TP White IR Crowther MJ. Using simulation studies to evaluate statistical methods. Stat Med 2019;38:2074–102. 30652356 10.1002/sim.8086 PMC6492164

[13] National Research Council. The prevention and treatment of missing data in clinical trials. Washington, DC: National Academies Press, 2010. 24983040

[14] Singla NK Desjardins PJ Chang PD. A comparison of the clinical and experimental characteristics of four acute surgical pain models: dental extraction, bunionectomy, joint replacement, and soft tissue surgery. PAIN 2014;155:441–56. 24012952 10.1016/j.pain.2013.09.002

[15] Singla NK Meske DS Desjardins PJ. Exploring the interplay between rescue drugs, data imputation, and study outcomes: conceptual review and qualitative analysis of an acute pain data set. Pain Ther 2017;6:165–75. 28676997 10.1007/s40122-017-0074-5 PMC5693805

[16] Stensrud MJ Dukes O. Translating questions to estimands in randomized clinical trials with intercurrent events. Stat Med 2022;41:3211–28. 35578779 10.1002/sim.9398 PMC9321763

[17] Suri P Heagerty PJ Korpak A Jensen MP Gold LS Chan KCG Timmons A Friedly J Jarvik JG Baraff A. Improving power and accuracy in randomized controlled trials of pain treatments by accounting for concurrent analgesic use. J Pain 2023;24:332–44. 36220482 10.1016/j.jpain.2022.09.017 PMC9898095

[18] Suri P Heagerty PJ Timmons A Jensen MP. Description and initial validation of a novel measure of pain intensity: the Numeric Rating Scale of underlying pain without concurrent analgesic use. PAIN 2024;165:1482–92. 38189184 10.1097/j.pain.0000000000003150 PMC11189761

[19] U.S. Food and Drug Administration. E9(R1) statistical principles for clinical trials: addendum: estimands and sensitivity analysis in clinical trials: guidance for industry. Available at: https://www.fda.gov/media/148473/download. Accessed November 12, 2025.

[20] U.S. Food and Drug Administration. Development of non-opioid analgesics for acute pain: Guidance for industry. Available at: https://www.fda.gov/media/156063/download. Accessed November 12, 2025.

[21] Viscusi ER de Leon-Casasola O Cebrecos J Jacobs A Morte A Ortiz E Sust M Vaque A Gottlieb I Daniels S Gimbel JS Muse D Winkle P Kuss ME Videla S Gascon N Plata-Salaman C. Celecoxib-tramadol co-crystal in patients with moderate-to-severe pain following bunionectomy with osteotomy: a phase 3, randomized, double-blind, factorial, active- and placebo-controlled trial. Pain Pract 2023;23:8–22. 35686380 10.1111/papr.13136 PMC10084286

[22] Wallace MS. Trials for managing acute pain—a clinically meaningful small effect size? N Engl J Med 2023;389:464–5. 37530828 10.1056/NEJMe2305480

[23] White IR Bamias C Hardy P Pocock S Warner J. Randomized clinical trials with added rescue medication: some approaches to their analysis and interpretation. Stat Med 2001;20:2995–3008. 11590628 10.1002/sim.927

