# Supplementary material for: Standard rescue medication adjustments produce biased estimates of analgesic efficacy: reducing bias with multiple imputation for rescue adjustment (MIRA)
Source: Pain. 2026 Jul 29;167(10):2439–51. doi: 10.1097/j.pain.0000000000004074 (PMC13580216; doi:10.1097/j.pain.0000000000004074)

Supplemental Digital Content

Standard rescue medication adjustments produce biased estimates of analgesic efficacy: reducing bias with multiple imputation for rescue adjustment (MIRA)

Christopher J. Miller[a], John T. Farrar[a]

a) Department of Biostatistics, Epidemiology, and Informatics, Perelman School of Medicine, University of Pennsylvania, Philadelphia, PA, United States

Rescue Drug Effect Estimation

Background

As described in the primary manuscript, CLCT-003 was used to estimate the analgesic effect of HC/APAP when used as a rescue pain medication. CLCT-003 was a double-blind, randomized, multiple-dose, placebo- and positive-controlled study of CL-108 (hydrocodone 7.5 mg/ acetaminophen [APAP] 325 mg, promethazine 12.5 mg) in participants with moderate to severe pain following bunionectomy (with osteotomy and internal fixation) without collateral procedures. A positive control (Norco®, a commercial formulation of hydrocodone 7.5 mg/APAP 325 mg) was included to determine the anti-emetic effects of CL-108.

Analgesia was determined by comparing CL-108 to placebo. To confirm baseline pain as moderate or severe, pain intensity was also measured on a 0-10 numerical pain intensity scale (NRS). Participants were required to have moderate (4-6) or severe pain (7-10) on the NRS in order to be eligible for randomization.

Qualifying patients were randomized to one of the three treatment groups in a 5:5:1 ratio: CL-108 (n = 252), Norco® (n = 250), or placebo (n = 50). Study medication was administered to patients under double-blind conditions. The same assigned study medication was administered for pain every 4-6 hours (for a maximum of 6 doses in a 24-hour period). Thus, participants received maximal doses (a total of 8-12 doses) of study medication over the first 48 hours.

Participants were also permitted to use supplemental analgesic medication (ibuprofen 400 mg) and/or anti-emetic medication as prescribed by the Investigator according to standard medical practice. Pain intensity was assessed on the PI-NRS at 10-minute time points over 2 hours after them first dose, then every 30 minutes over the next 4 hours.

Consistent with bunionectomy trials more generally, most participants were female (88%), and the median age was approximately 41 years (**Supplemental Table 1**). The median baseline pain score on the NRS scale was 7 (IQR, 5 to 8). Half of participants had a severe baseline pain score. All participants were enrolled in the US between 2014 and 2015.

Supplemental Table 1. Baseline characteristics in CLCT-003

Characteristic	HC/APAP/Promethazine 7.5/325/12.5 mg (N=252)	HC/APAP (N=250)	Placebo (N=50)
Female sex, n (%)	223 (88.5%)	222 (88.8%)	43 (86.0%)
Age, mean (SD)	41.2 (13.7)	41.7 (13.5)	41.8 (13.1)
Baseline NRS, mean (SD)	6.6 (1.7)	6.7 (1.7)	6.9 (1.8)
Baseline NRS ≥7, n (%)	127 (50.4%)	126 (50.4%)	25 (50.0%)
Enrolled in U.S., n (%)	252 (100%)	250 (100%)	50 (100%)

During the first 4-6 hour dosing interval, 72% of placebo-treated subjects took rescue vs 35-40% of those who took HC/APAP (**Supplemental Figure 1**). For defining the HC/APAP treatment effect, the HC/APAP arm with promethazine was not considered.

Supplemental Figure 1. Time to first use of rescue medication in CLCT003

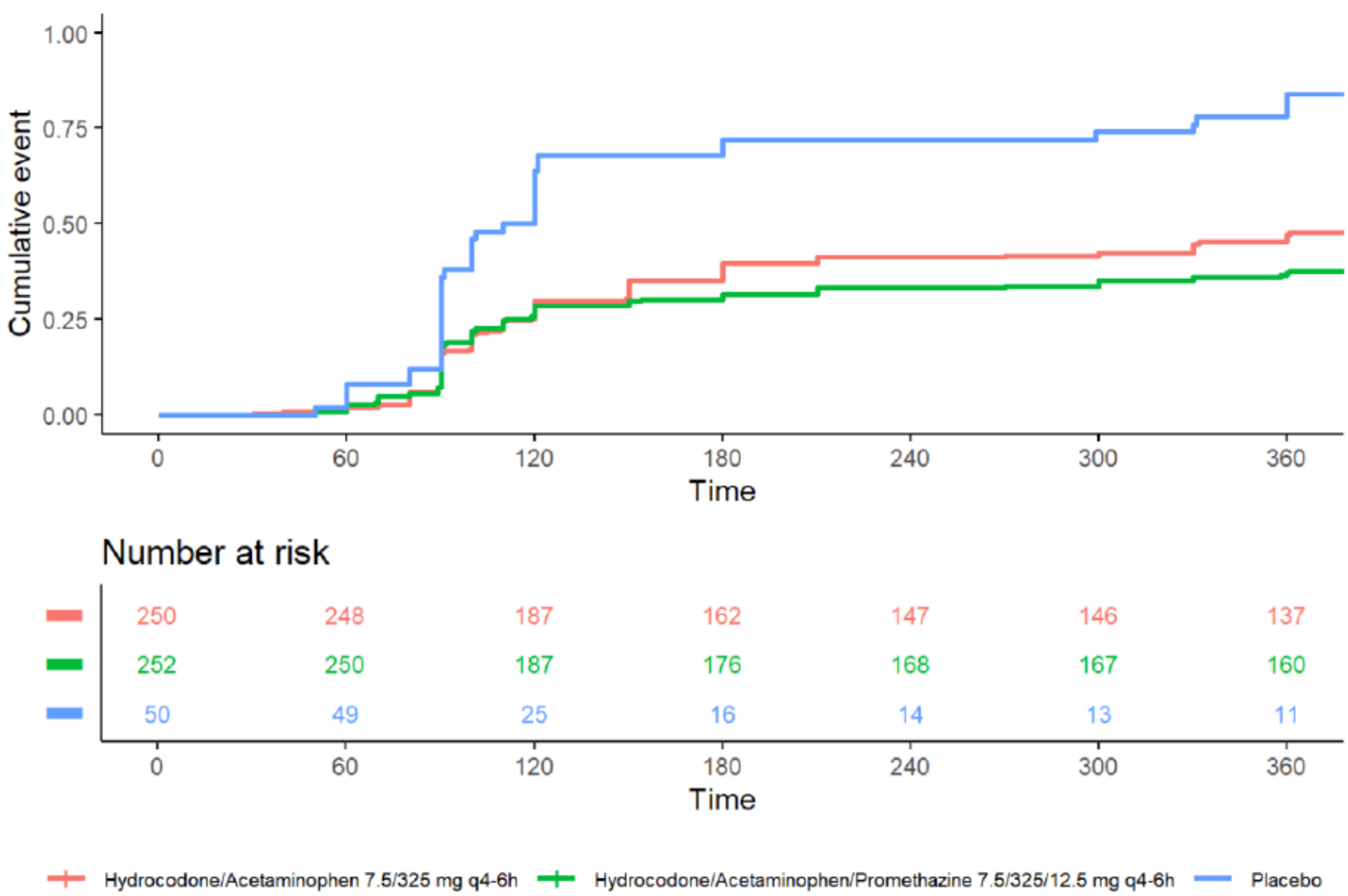

To explore the effect of rescue-taking within the first dosing interval, we evaluated the average treatment effect with no imputation, LOCF, BOCF, and WOCF imputation (**Supplemental Figure 2**). Given the increasing use of rescue in all groups, the mean differences are more

difficult to interpret as they reflect a combination of the effect of placebo, HC/APAP, and rescue medication.

Supplemental Figure 2. Treatment difference for HC/APAP in CLCT-003 by imputation method

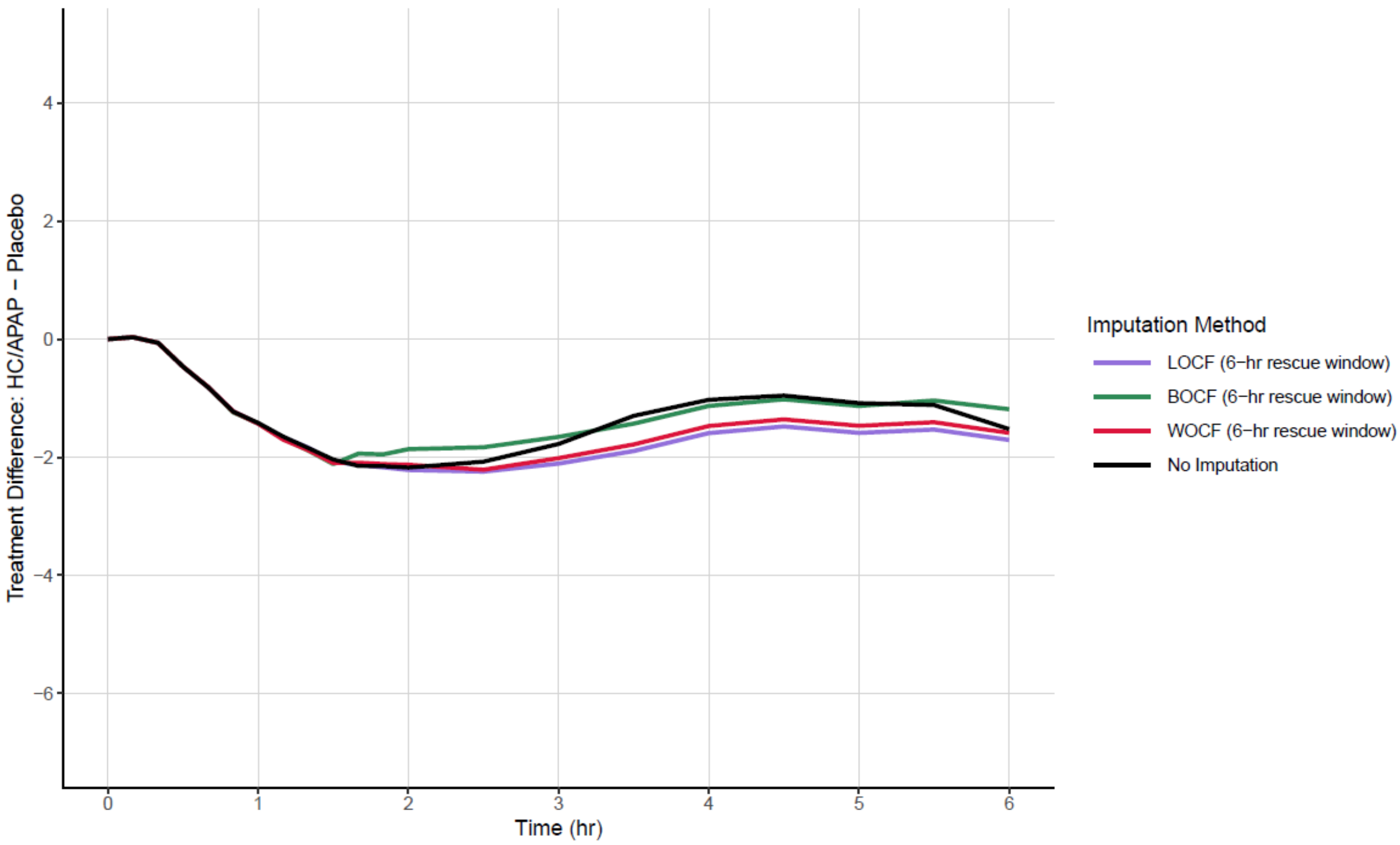

<u>Deriving the Reference-Based Distribution</u>

To incorporate the data-driven estimates from CLCT-003 with known pharmacologic information, we used a parametric onset-offset function, $f(t) = a \cdot t^b \cdot e^{-ct}$, to model the expected treatment effect profile with a 6-hour duration of action. This function naturally captures the characteristic pharmacodynamic profile of oral analgesics:

- a reoresents the magnitude scaling factor that determines the overall intensity of the treatment effect (fitted estimate: -5.304);
- b controls the rate of onset (fitted estimate: 2.631);
- c controls the elimination rate (fitted estimate: 1.344), and;
- t represents time.

This mathematical form is particularly well-suited because it constrains the model to produce a single-peaked, biologically plausible treatment effect profile that rises gradually from baseline, reaches a maximum effect, and then declines naturally toward zero, which is what would be expected from the known PK and PD properties of HC/APAP in the absence of confounding factors such as rescue medication use. This model was fit to all observed mean treatment differences in pain scores through 3 hours, whereafter we deemed the interference of rescue medication too great to reflect the true drug effect and the model assumptions extrapolated the return to no effect by 6 hours. The standard deviation of scores over time in the HC/APAP group were applied as the error to capture the national variability of individual participants within the HC/APAP group around their expected trajectory (**Supplemental Figure 3**). Therefore, our approach assumes that participants who take HC/APAP as a rescue medication will experience the placebo-adjusted analgesic benefit described by the fitted curve, with individual variation consistent with what was observed among participants originally randomized to receive HC/APAP in the same surgical model. The estimated parameters for this reference distribution are provided in **Supplemental Table 2**.

Supplemental Figure 3. Fitted parametric onset-offset curve to CLCT-003 data

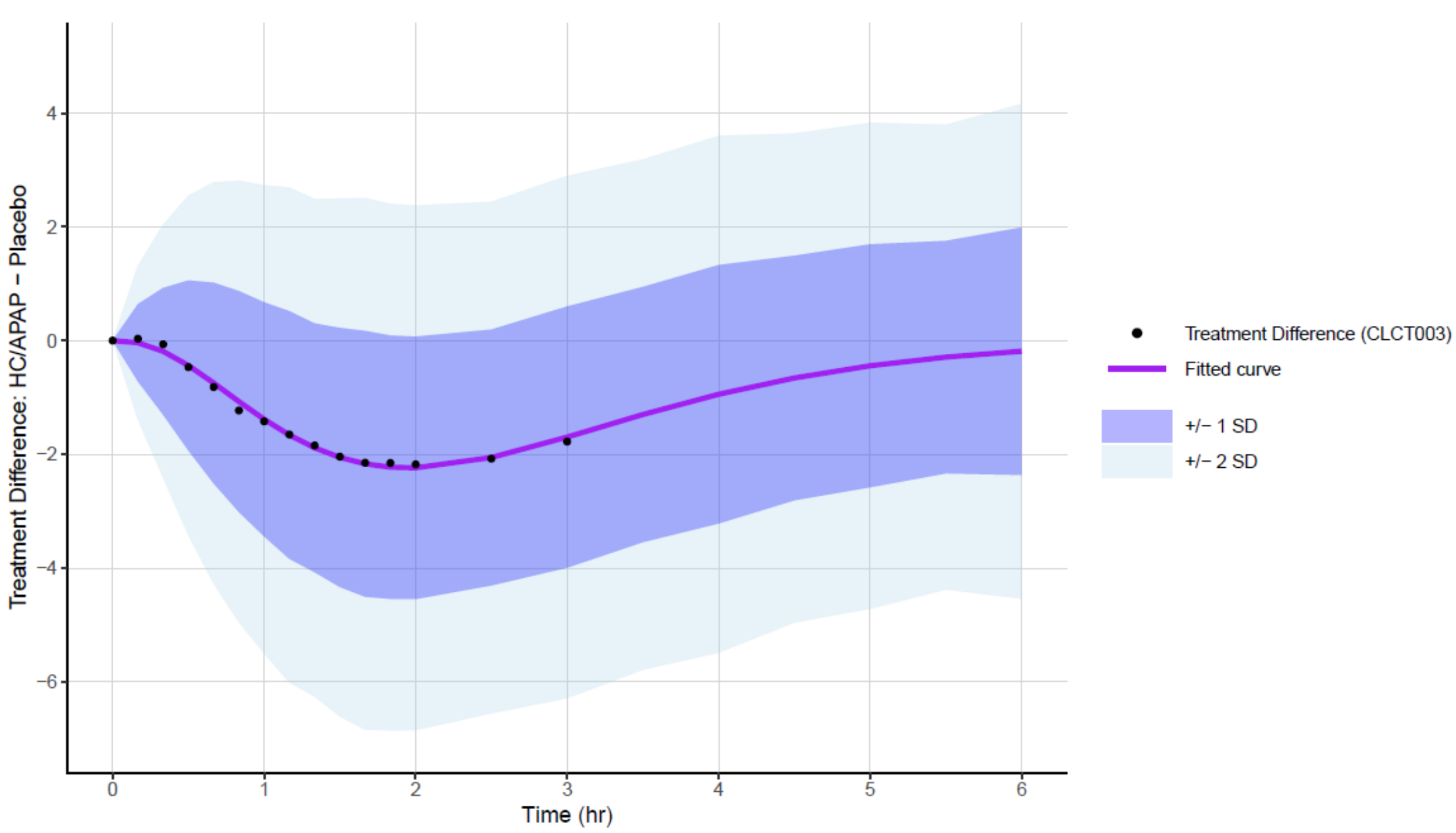

Supplemental Table 2. Estimated mean treatment effect values from the parametric onset-offset model with standard deviations based on empirical variability in HC/APAP group of CLCT-003

Time (min)	Time (hr)	Estimated mean treatment effect (NRS scale)	SD (HC/APAP group)
0	0.0	0.000	0.000
10	0.167	-0.038	0.679
20	0.333	-0.188	1.114
30	0.5	-0.437	1.495
40	0.667	-0.745	1.764
50	0.833	-1.072	1.942
60	1.0	-1.384	2.058
70	1.167	-1.660	2.177
80	1.333	-1.885	2.187
90	1.5	-2.054	2.278
100	1.667	-2.167	2.337
110	1.833	-2.226	2.315
120	2.0	-2.237	2.307
150	2.5	-2.056	2.249
180	3.0	-1.696	2.296
210	3.5	-1.300	2.244
240	4.0	-0.944	2.272
270	4.5	-0.657	2.150
300	5.0	-0.443	2.137
330	5.5	-0.291	2.044
360	6.0	-0.187	2.176

Diagnostic Analyses

We conducted a set of exploratory diagnostic analyses on the CLCT-003 reference data to evaluate the assumptions underlying the use of the HC/APAP reference distribution. We examined whether the placebo-adjusted rescue effect would reasonably be modeled as additive or multiplicative on the NRS scale, and we examined whether the placebo-adjusted rescue effect varied meaningfully by (baseline) pain intensity, age, and sex that could differ between the reference trial and a target trial and threaten transportability.

Model Specification

To explore the effects of HC/APAP and placebo following bunionectomy without the confounding influence of rescue medication, pain data from CLCT-003 were censored after first rescue use. We fit subject-level random-intercept generalized additive models (GAMs) to model

NRS pain scores. GAMs incorporate flexible, group-specific smooth time trends while accounting for within-subject correlation through a random intercept; this combination retains nonparametric flexibility while reducing bias from informative censoring induced by rescue use. The GAM models were parameterized as:

$$Y_{ij} = \alpha_{g[i]} + f_{g[i]}(t_{ij}) + b_i + \epsilon_{ij}$$

where Y_{ij} is the NRS pain score for subject i at time t_{ij}, $g[i]$ is the treatment group for subject i, $\alpha_{g[i]}$ is the group-specific intercept, $f_{g[i]}(t_{ij})$ is the group-specific smooth function of time, $b_i \sim N(0, \sigma_b^2)$ is the subject-level random intercept and $\epsilon_i \sim N\ 0, \sigma_b^2$ is the residual error.

For each diagnostic analysis we fit separate GAMs within strata of the variable of interest (baseline pain intensity, age, or sex) and compared the modeled change in NRS over time within each treatment group and the modeled placebo-adjusted treatment-effect curve (HC/APAP-placebo) across strata. Treatment-effect curves that are similar across strata of baseline pain intensity support an additive specification of the rescue effect, while treatment-effect curves that are similar across age and sex strata support the use of a common reference distribution across these participant characteristics.

Analyses by Baseline Pain Intensity

Participants were stratified by baseline pain intensity (moderate 4 to <7 vs severe ≥7) and fit separate GAMs within each stratum for the HC/APAP and placebo groups (**Supplemental Figure 4**). The placebo-adjusted treatment effect curve was obtained as the difference of the group-specific smooth functions within each stratum (**Supplemental Figure 5**). Although absolute changes from baseline were larger in the severe baseline pain stratum, the placebo-adjusted treatment effect was similar across strata for approximately 200 minutes. After that time, the placebo group was heavily censored by rescue use and results are less reliable (**Supplemental Table 3**). We interpret the lack of a meaningful difference in the HC/APAP effect by baseline pain as supportive of the additivity assumption for the rescue effect within dosing intervals.

Supplemental Figure 4. Change in NRS by treatment group fitted by GAMs stratified by baseline pain during first dosing interval in CLCT-003 with participants censored at first rescue

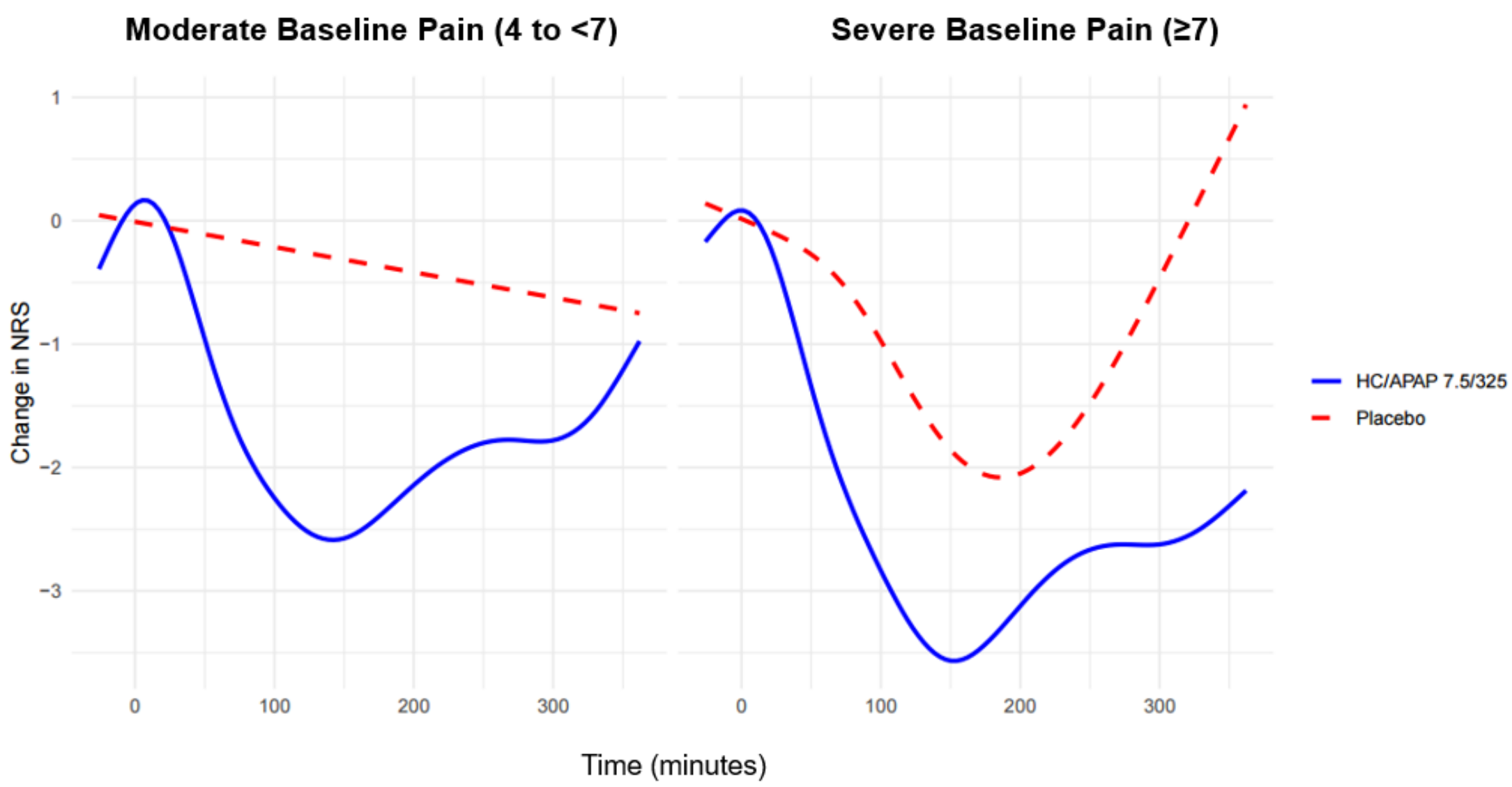

Supplemental Figure 5. Placebo-adjusted treatment effect curves fitted by GAMs stratified by baseline pain during first dosing interval in CLCT-003 with participants censored at first rescue

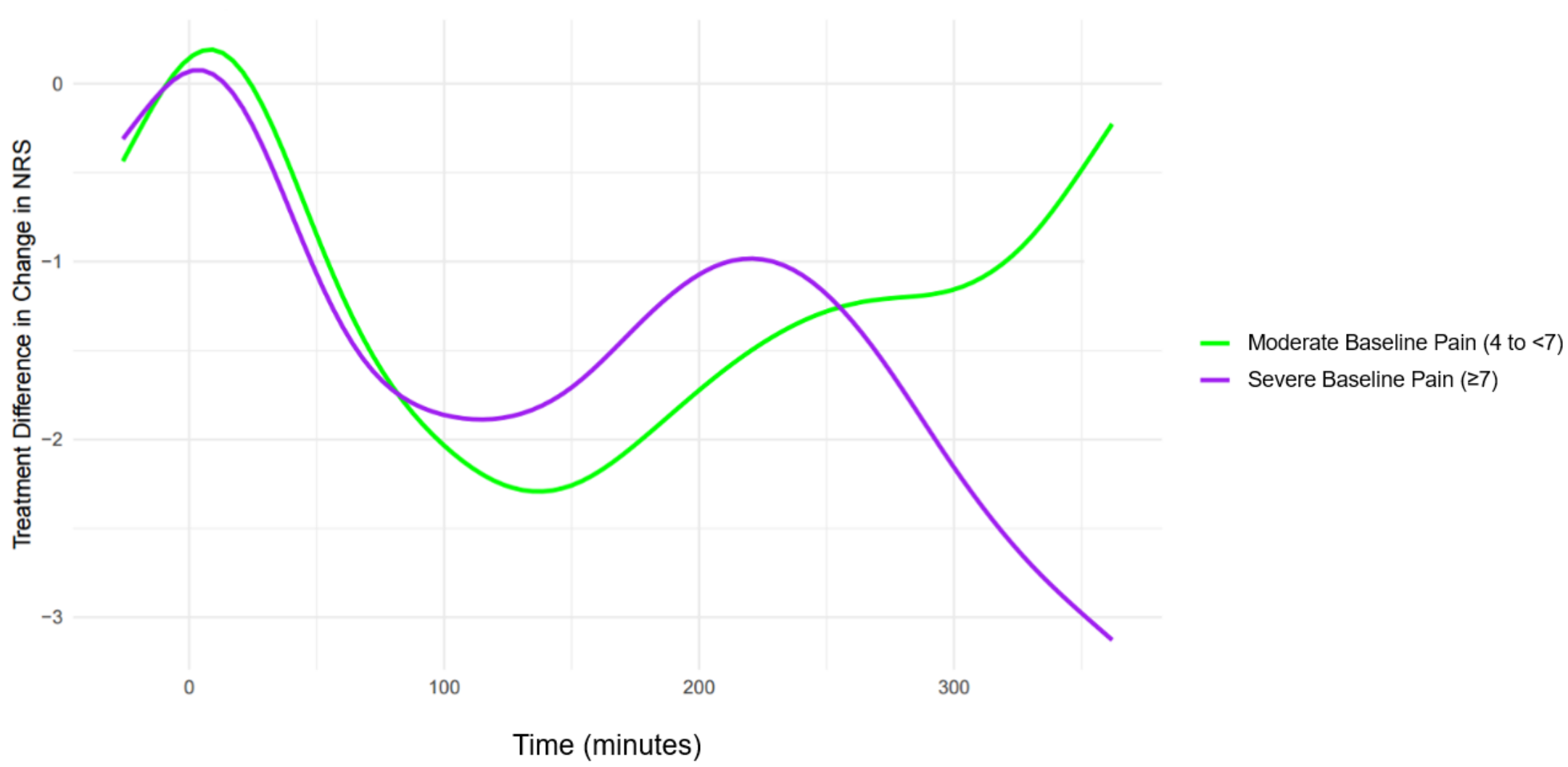

Supplemental Table 3. Number of participants contributing observations at each scheduled time point, by baseline pain stratum and treatment group

Number of Participant Included in Analysis (at risk)							
**Baseline Pain**	**Group**	**Time (minutes)**					
		**0**	**60**	**120**	**180**	**240**	**300**
Moderate (4 to <7)	HC/APAP	249	248	213	195	170	108
	Placebo	25	25	17	11	9	5
Severe (≥7)	HC/APAP	253	250	160	142	131	65
	Placebo	25	24	8	5	4	2

Analyses by Age

Participants were stratified at <40 versus ≥40 years, and the GAM analysis was repeated. The changes from baseline were similar for HC/APAP and placebo across age strata (**Supplemental Figure 6**), and the resulting placebo-adjusted treatment effect curves were correspondingly similar throughout most of the dosing interval (**Supplemental Figure 7**). The divergence of curves at later points occurred after the placebo group had been substantially censored by rescue use (**Supplemental Table 4**).

Supplemental Figure 6. Change in NRS by treatment group fitted by GAMs stratified by age during first dosing interval in CLCT-003 with participants censored at first rescue

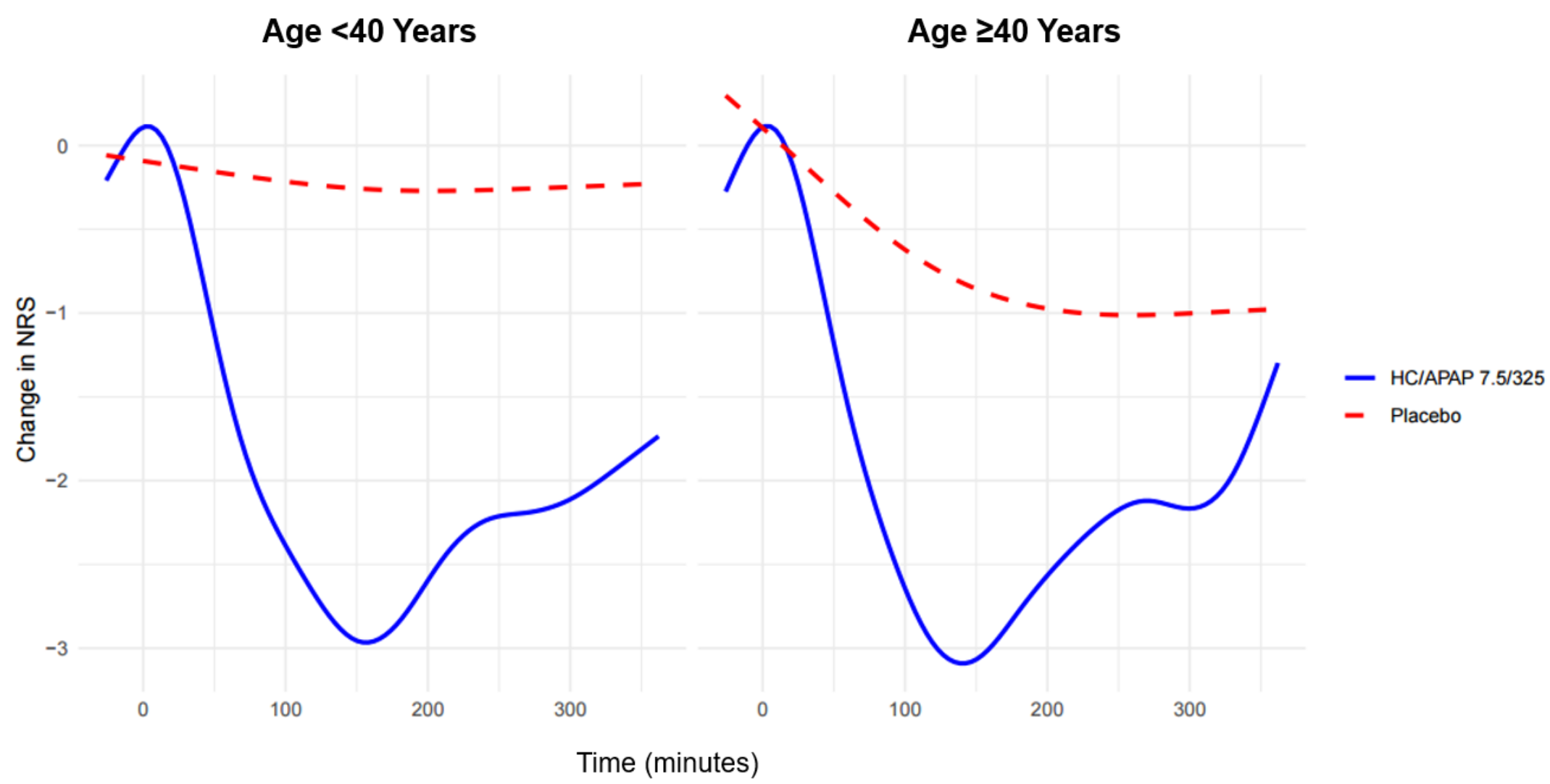

Supplemental Figure 7. Placebo-adjusted treatment effect curves fitted by GAMs stratified by age during first dosing interval in CLCT-003 with participants censored at first rescue

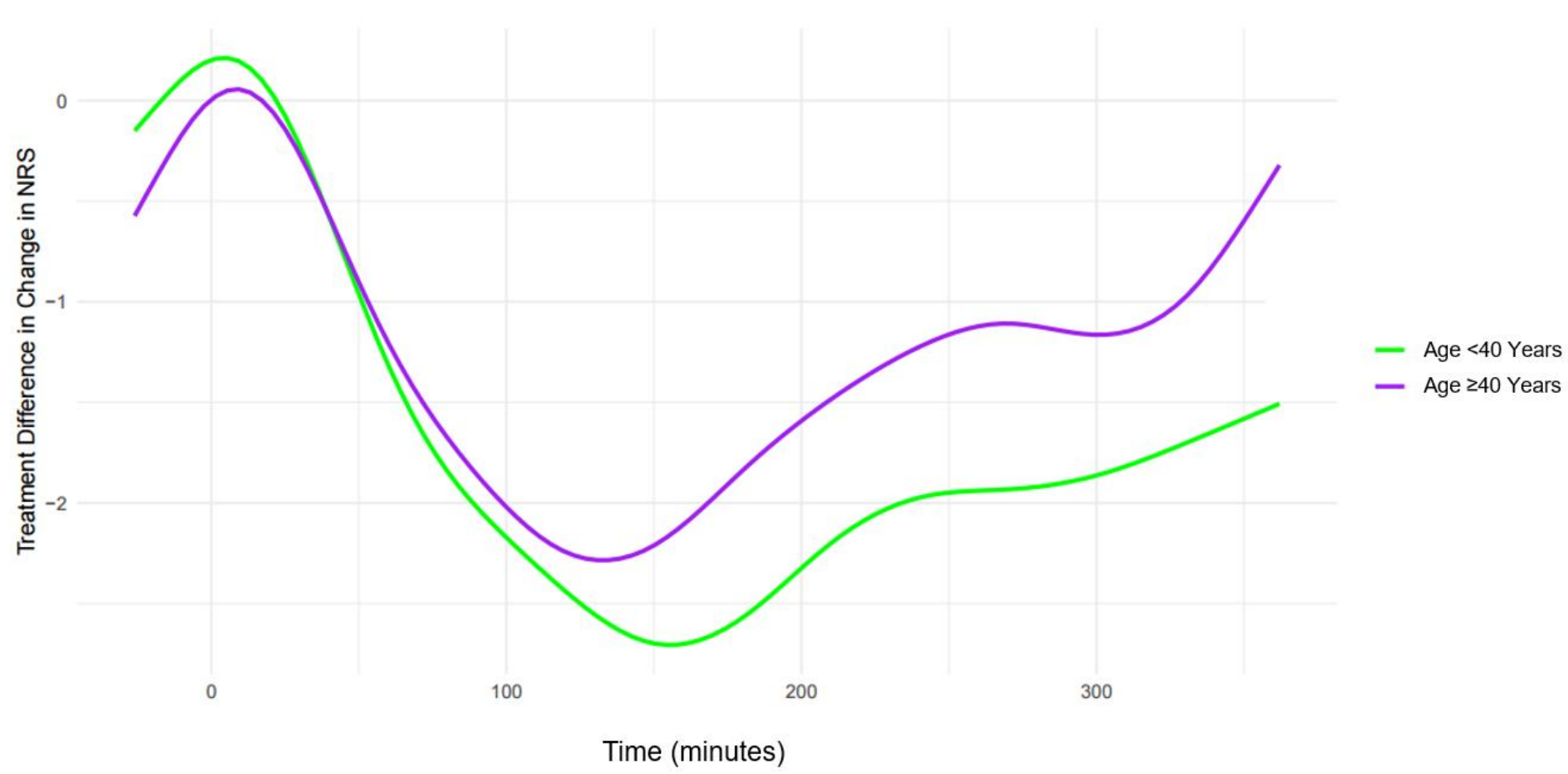

Supplemental Table 4. Number of participants contributing observations at each scheduled time point, by age stratum and treatment group

Number of Participant Included in Analysis (at risk)							
**Age**	**Group**	**Time (minutes)**					
		**0**	**60**	**120**	**180**	**240**	**300**
<40 Years	HC/APAP	220	218	165	148	135	77
	Placebo	22	21	8	4	4	2
≥40 Years	HC/APAP	282	280	208	189	166	96
	Placebo	28	28	17	12	9	5

Analyses by Sex

Sex was challenging to evaluate because of the small number of male participants in CLCT-003 (**Supplemental Table 5**), which is typical of bunionectomy trials more generally. With this caveat, the diagnostic plots showed no meaningful heterogeneity by sex except for a suggestion of a stronger placebo effect in females (**Supplemental Figure 8**). The placebo-adjusted treatment effect curves were similar in onset, peak and magnitude in the first dosing interval (**Supplemental Figure 9**).

Supplemental Figure 8. Change in NRS by treatment group fitted by GAMs stratified by sex during first dosing interval in CLCT-003 with participants censored at first rescue

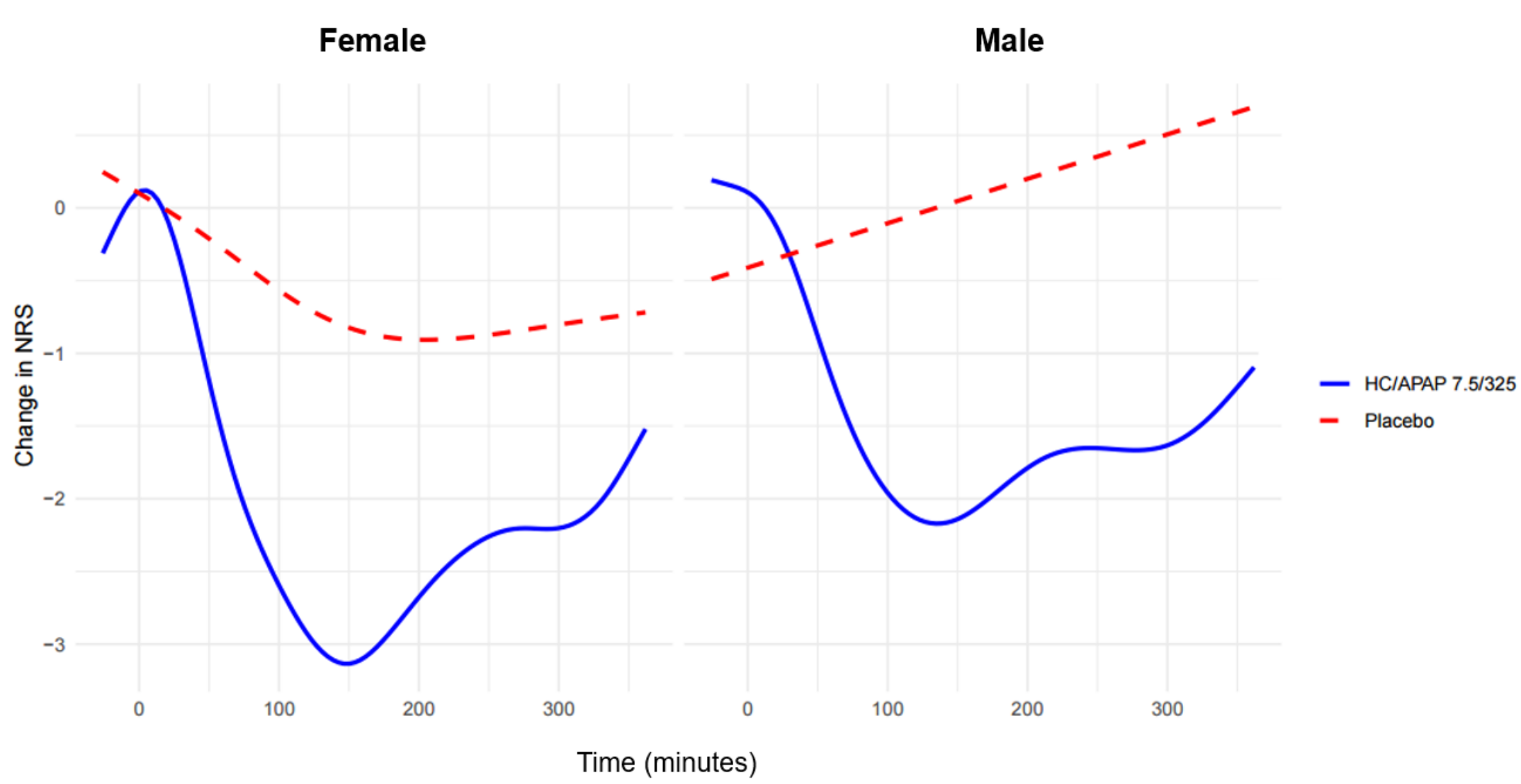

Supplemental Figure 9. Placebo-adjusted treatment effect curves fitted by GAMs stratified by sex during first dosing interval in CLCT-003 with participants censored at first rescue

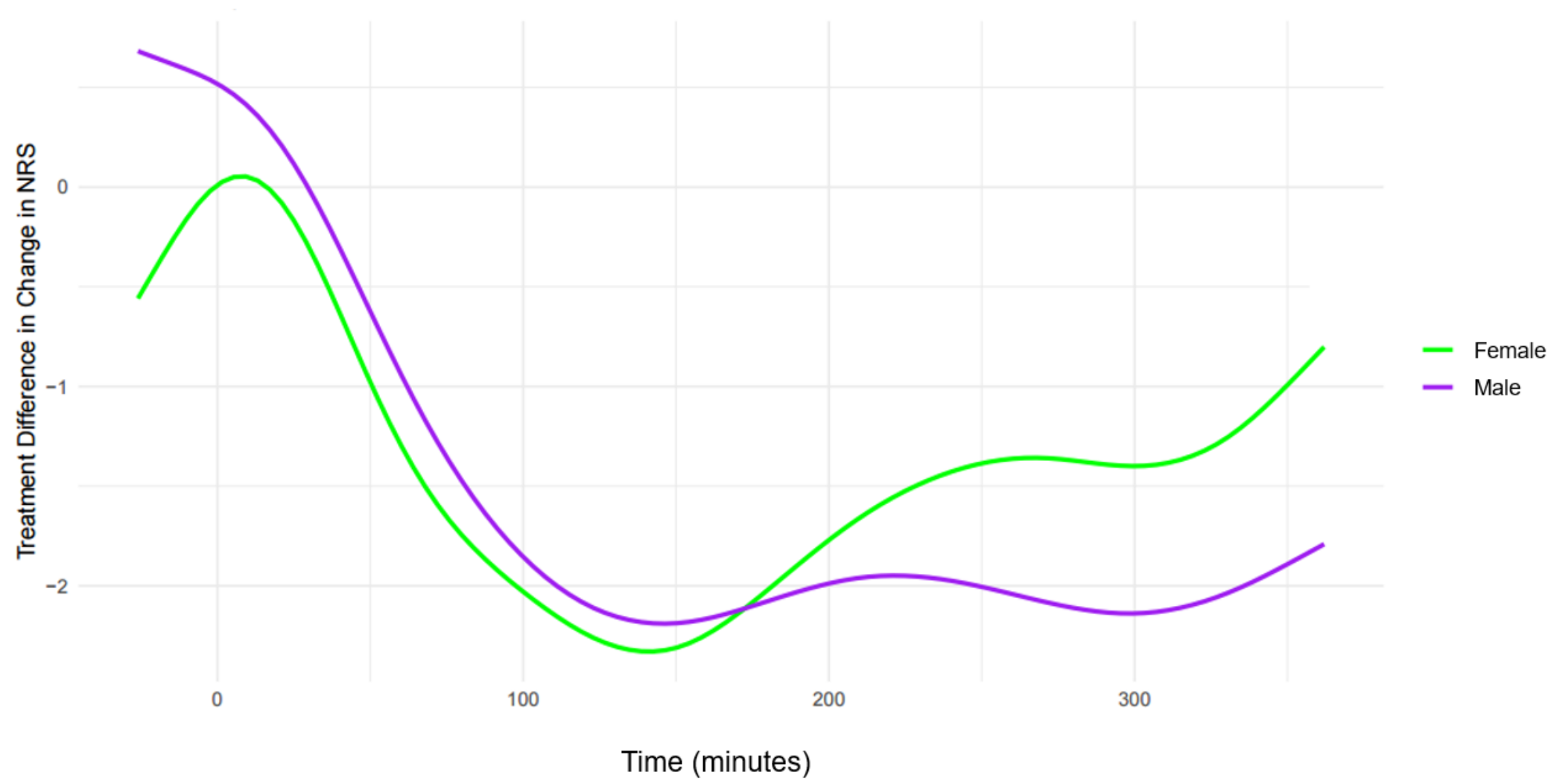

Supplemental Table 5. Number of participants contributing observations at each scheduled time point, by sex and treatment group

Number of Participant Included in Analysis (at risk)							
**Sex**	**Group**	**Time (minutes)**					
		**0**	**60**	**120**	**180**	**240**	**300**
Female	HC/APAP	445	441	332	299	265	152
	Placebo	43	42	22	15	12	6
Male	HC/APAP	57	57	41	38	36	21
	Placebo	7	7	3	1	1	1

Counterfactual Parameter Estimation

<u>Background</u>

We sought to estimate counterfactual pain intensity scores over time using the individual participant data from the placebo arms of bunionectomy trials, focusing on oral administration of study drugs administered every 6 hours, a common dosing interval for oral analgesics. The compiled database of Phase 2 and 3 trials previously submitted to the FDA has been described in detail previously [9]. Of all 46 trials in the database, there were 13 oral bunionectomy trials. Three trials used a strict 6-hour dosing over a 48-hour period: AP-ADF-105 (oxycodone/niacin), ELI-200-003-2014 (oxycodone/naltrexone), and ESTEVE-SUSA-301 (celecoxib/tramadol co-crystal and individual components).

The baseline characteristics of the placebo arms of the three studies were generally similar (**Supplemental Table 6**). More than 80% of participants were female in each trial, the average age ranged from 40 to 46, and all participants were enrolled in the U.S. Mean pain scores were between 6.4 and 7.5 in the placebo groups at baseline, and ELI-200-003-2014 had a higher proportion of participants with severe pain at baseline (70%) than the other trials (45% or 53%).

Supplemental Table 6. Baseline characteristics in placebo groups of AP-ADF-105, ELI-200-003-2014, and ESTEVE-SUSA-301

**Characteristic**	**AP-ADF-105 Placebo (N=136)**	**ELI-200-003-2014 Placebo (N=53)**	**ESTEVE-SUSA-301 Placebo (N=89)**
Female sex, n (%)	114 (83.8%)	45 (84.9%)	75 (84.3%)
Age, mean (SD)	42.0 (13.0)	40.3 (13.8)	46.1 (14.9)
Baseline NRS, mean (SD)	6.4 (1.99)	7.5 (1.81)	6.8 (1.30)
Baseline NRS ≥7, n (%)	61 (44.9%)	37 (69.8%)	47 (52.8%)
Enrolled in U.S., n (%)	136 (100%)	53 (100%)	89 (100%)

More than 75% of participants in the placebo group of each trial took rescue medication within the first 6 hours (**Supplemental Figure 10**). At least a quarter of placebo participants had taken rescue at any 6-hour interval. The fact that pain intensity scores are affected by the use of rescue medication by an appreciable proportion of participants in the placebo group throughout the trials likely increased the variability in responses observed, which was accounted for in the selection of the simulation parameters.

Supplemental Figure 10. Proportion of placebo participants taking rescue by time and trial

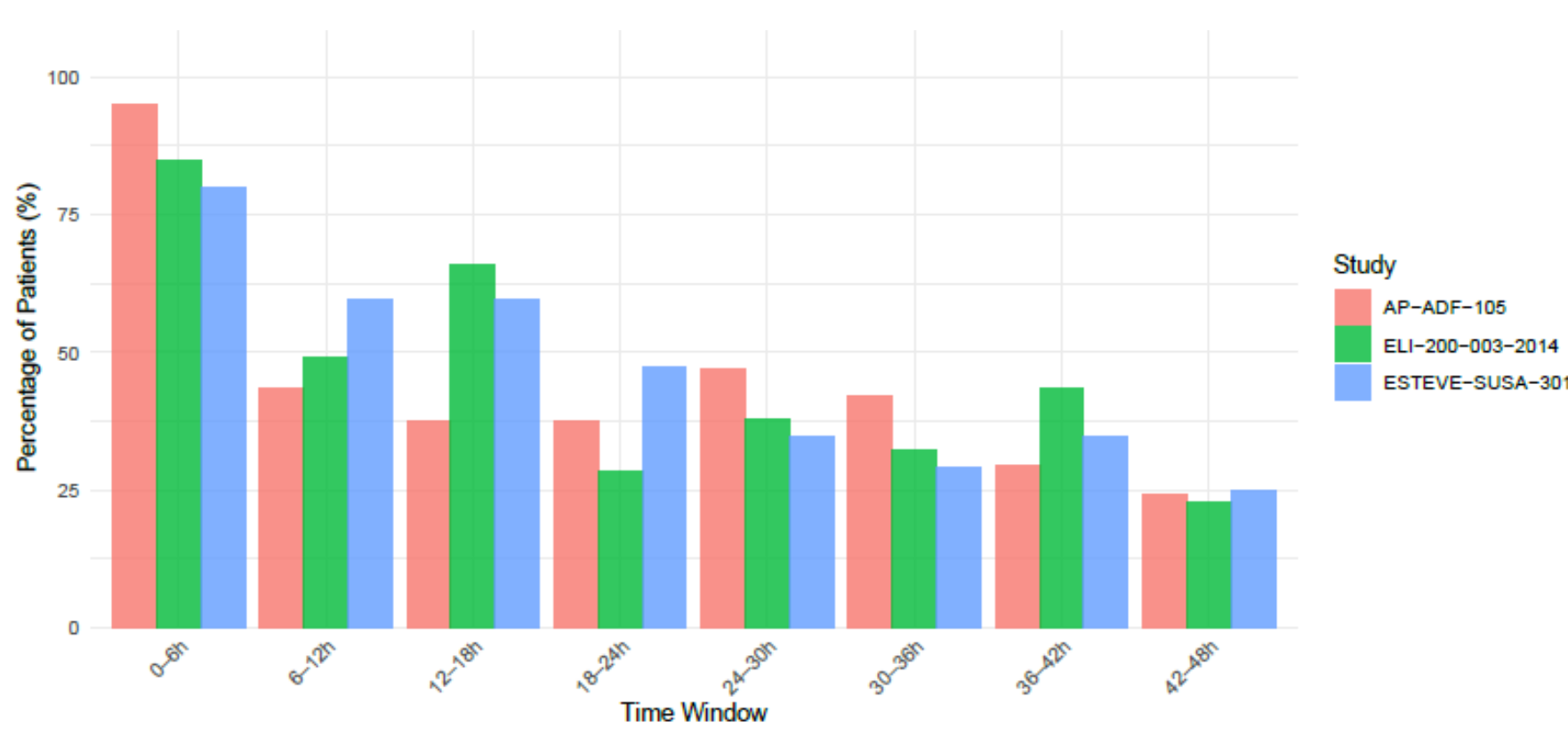

Baseline and Endpoint Pain Distributions

Baseline pain intensity ranged from 6.3 to 7.3 (SD, 1.3 to 2.1) across the trials (**Supplemental Table 7**). Observed 48-hour pain scores ranged from 2.4 to 2.9, however rescue medication use was substantial throughout the trial period as previously described. Given this high rescue utilization, observed endpoint pain scores (at 48 hours) likely underestimate counterfactual pain in the absence of rescue medication. Based on observed trajectories and rescue patterns, we selected a counterfactual mean 48-hour pain score of 4.0 (SD, 2.3) for simulation purposes.

Supplemental Table 7. Mean and standard deviation of pain intensity scores (NRS scale) in placebo groups at baseline and 48 hours

Study ID	N	Hour	Mean	SD
AP-ADF-105	136	0	6.4	1.99
		48	2.4	2.12
ELI-200-003-2014	53	0	7.5	1.81
		48	2.9	2.15
ESTEVE-SUSA-301	89	0	6.8	1.30
		48	2.4	1.97

Between-Individual Variability in Pain Trajectories

The cross-sectional standard deviation of pain intensity remained relatively stable over scheduled time points across all 3 trials, ranging from 2.0 to 2.6 throughout the 48-hour period (**Supplemental Figure 11**). This consistency in population-level variability supported the use of time-invariant between-individual variance parameters in the simulation.

Supplemental Figure 11. Standard deviation of pain intensity scores (NRS scale) at scheduled time points by trial

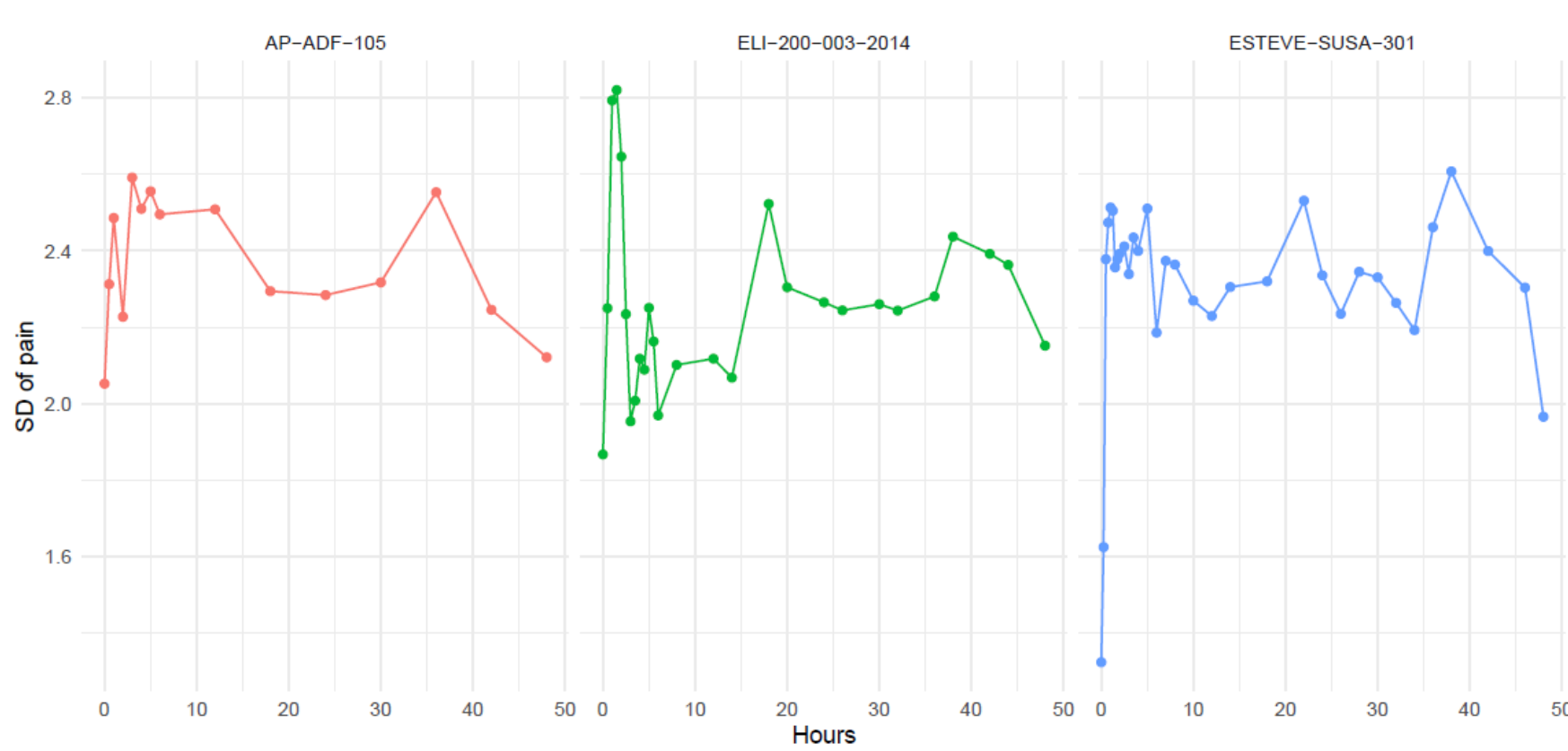

Pain Trajectory Slopes

To characterize pain progression, we estimated individual-level linear slopes during two periods: early post-baseline (0-6 hours) and after the initial re-dosing (6-48 hours).

For early slopes (0-6 hours), mean slopes range from -0.14 to -0.31 NRS points per hour across studies (SD, 0.47 to 0.54), reflecting an initial decline in pain following the first dose (**Supplemental Table 8**). There was a moderate negative correlation (range, -0.29 to -0.56) between baseline pain intensity and early slope, indicating the participants with more severe baseline pain experienced steeper early improvements (**Supplemental Table 9**).

After the first re-dosing (6-48 hours), mean slopes were more modest, ranging from -0.048 to -0.078 NRS points per hour (SD, 0.06 to 0.18), reflecting a slower rate of continued improvement after re-dosing with placebo (**Supplemental Table 8**). These slope estimates informed the systematic trajectory components in our counterfactual simulations.

Supplemental Table 8. Summary statistics for early (0-6 hours) and late (6-48 hours) slopes in placebo groups

Study ID	Mean	SD	Median	Q25	Q75
AP-ADF-105					
Early slope (0-6 hr)	-0.309	0.537	-0.323	-0.580	-0.074
Late slope (6-48 hr)	-0.048	0.060	-0.038	-0.082	-0.007
ELI-200-003-2014					
Early slope (0-6 hr)	-0.140	0.470	-0.22	-0.363	0.077
Late slope (6-48 hr)	-0.078	0.064	-0.067	-0.107	-0.031
ESTEVE-SUSA-301					
Early slope (0-6 hr)	-0.299	0.498	-0.274	-0.546	-0.028
Late slope (6-48 hr)	-0.054	0.178	-0.072	-0.105	-0.034

Supplemental Table 9. Correlation between baseline pain scores and early and late slopes in placebo groups

Study ID	Baseline-Early Slope Correlation	Baseline-Late Slope Correlation
AP-ADF-105	-0.294	-0.295
ELI-200-003-2014	-0.557	-0.351
ESTEVE-SUSA-301	-0.341	0.122

Within-Individual Correlation Structure

Pain scores measured at adjacent timepoints in the same individual are not independent. To generate realistic temporal dependencies in pain scores for simulated individuals over time, we examined autocorrelation patterns in each trial. Autocorrelation functions demonstrated rapid decay after a single lag (i.e., scheduled pain assessments that were not immediately next to one another), supporting a first-order autoregressive [AR(1)] correlation structure (**Supplemental Figure 12**).

Supplemental Figure 12. Average within-subject autocorrelation function of residuals by trial

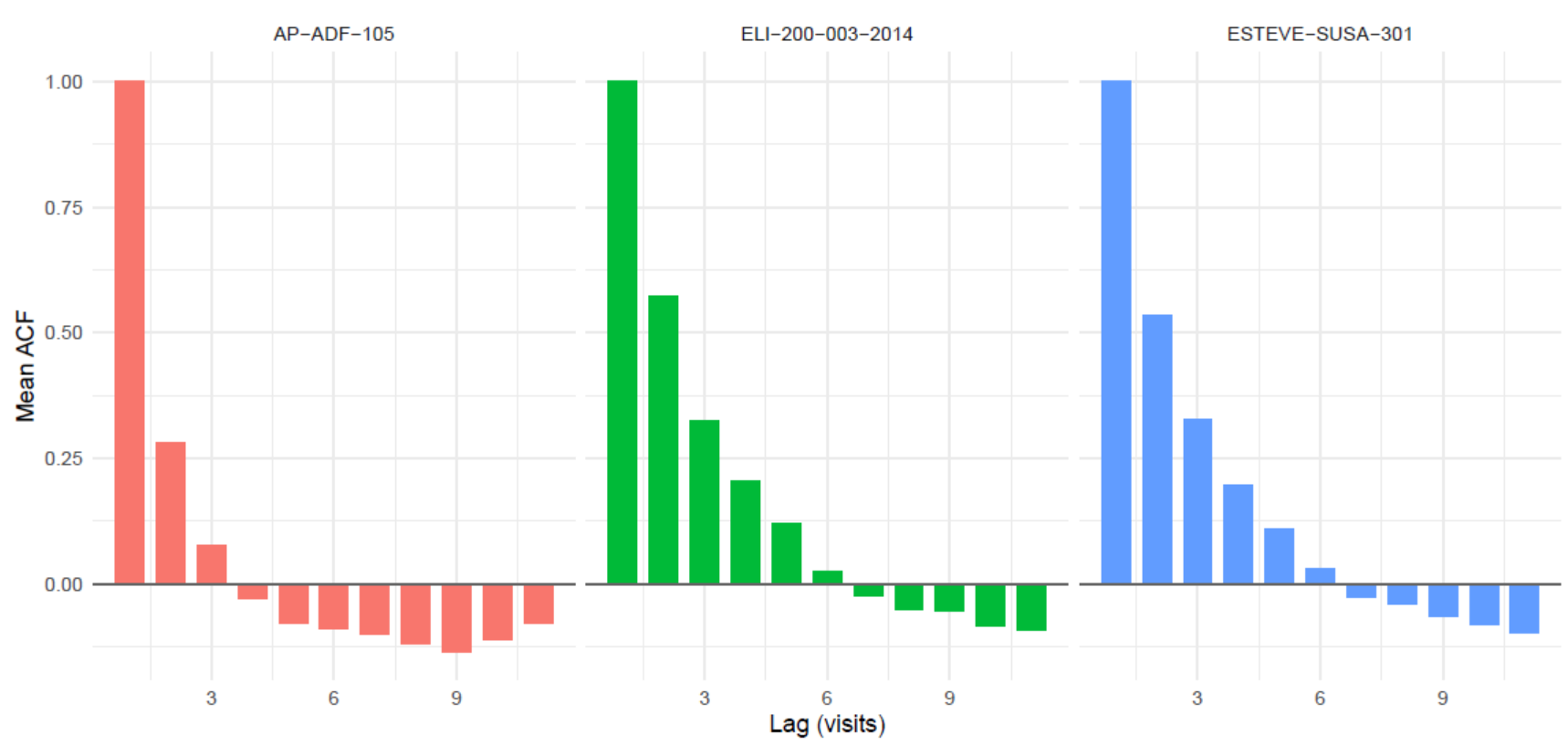

To estimate AR(1) parameters, we first removed population-level time trends using a generalized additive model, which models pain as a function of time without imposing a specific distribution. This model allowed us to decompose population trajectories from individual-level deviations. The lag-1 correlations of individual residuals yielded median AR(1) coefficients of 0.64 and 0.65 for ELI-200-003-2014 and ESTEVE-SUSA-301, respectively. AP-ADF-105 showed a lower median correlation of 0.37, which was likely attributable to its sparser assessment schedule (**Supplemental Table 10**). These coefficients were used to generate temporally correlated within-individual variation in pain scores in the simulations.

Supplemental Table 10. Summary statistics for AR(1) correlations by trial

Study ID	N	Mean	Median	Q25	Q75
AP-ADF-105	135	0.328	0.370	0.044	0.615
ELI-200-003-2014	53	0.610	0.644	0.524	0.755
ESTEVE-SUSA-301	89	0.567	0.649	0.413	0.757

Residual Variability

The average standard deviation of GAM residuals, representing within-individual variability after accounting for population trends and autocorrelation, ranged from 1.52–1.68 across studies (**Supplemental Table 11**). These residual standard deviations informed the magnitude of fluctuations from scheduled pain assessment to assessment around each individual's expected trajectory. As previously indicated, these estimates likely represented upper bounds on true within-individual counterfactual variability since the observed data from the 3 trials includes additional noise from rescue medication effects.

Supplemental Table 11. Summary statistics for residuals from generalized additive model

Study ID	Mean Standard Deviation of Residuals	Median Standard Deviation of Residuals
AP-ADF-105	1.68	1.67
ELI-200-003-2014	1.52	1.52
ESTEVE-SUSA-301	1.58	1.47

Details on Counterfactual Simulation

The counterfactual parameters estimated above were integrated into a framework for simulating individual participant pain trajectories in bunionectomy trials with 6-hour oral dosing. The simulation combines deterministic mean trajectories for treatment groups with stochastic individual variation to generate realistic pain score time serious that capture both treatment effects and natural recovery patterns that were observed in historical trials. Counterfactual pain trajectories $Y_i\ t$ for individuals are generated using the model:

$$Y_i\ t\ = \mu\ t\ + [\alpha_i + \beta_i \cdot t] + \epsilon_i(t)$$

where $\mu\ t$ is the population mean trajectory, $\alpha_i + \beta_i\ t$ are individual-specific random effects, and $\epsilon_i(t)$ controls the within-individual temporal correlation and residual error. Simulated scores are rounded to integers and bounded to the support of the NRS [0, 10].

Population Mean Trajectory

The population mean pain trajectory combines the natural recovery process plus treatment-specific dosing effects:

$$\mu\ t\ = \mu_{\text{base}}\ t\ + \mu_{\text{dose}}\ t$$

The base recovery process follows an exponential approach from baseline to 48 hours:

$$\mu_{\text{base}}\ t\ = \mu_{48} + (\mu_0 - \mu_{48}) \times (1 - e^{-\lambda t})$$

where μ_0 is the baseline mean pain, μ_{48} is the mean pain at 48 hours, λ is the decay rate, and t is the time (in hours). While the exponential form captures relatively rapid improvement in the first 12 hours and gradual continued improvement through 48 hours, prior studies suggested more profound dose effects with distinct onset and offset phases with the first and second administrations of study drug, which were not captured by a simple exponential curve. To capture these unique aspects of the q6h dosing regimen, dose effects $\mu_{dose}\ t$ were applied over the first 12 hours with doses administered at $t = 0$ and $t = 6$ hours:

For the first dose effects, $t \in (0{,}6]$ hours:

$$D_1(t) = \begin{cases} -\delta_1 \times \dfrac{t}{1.5} & \text{if } 0 < t \leq 1.5 \text{ (onset phase)} \\ -\delta_1(1-r) - \delta_1 r \left(1 - \dfrac{t-1.5}{4.5}\right) & \text{if } 1.5 < t \leq 6 \text{ (offset phase)} \end{cases}$$

For the second dose effects, $t \in (6{,}12]$ hours, letting $t^* = t - 6$:

$$D_2(t) = \begin{cases} -\delta_2 \times \dfrac{t^*}{1.5} & \text{if } 0 < t^* \leq 1.5 \text{ (onset phase)} \\ -\delta_2(1-r) - \delta_2 r \left(1 - \dfrac{t^*-1.5}{4.5}\right) & \text{if } 1.5 < t^* \leq 6 \text{ (offset phase)} \end{cases}$$

where δ_1 is the maximum pain reduction from the first dose, δ_2 is the maximum pain reduction from the second dose, and r is the proportion of the rebound effect (i.e., the degree to which the dosing effect wanes during the offset). Therefore, the total dosing effect is:

$$\mu_{\text{dose}}(t) = \begin{cases} D_1(t) & \text{if } 0 < t \leq 6 \\ D_2(t) & \text{if } 6 < t \leq 12 \\ 0 & \text{if } t > 12 \end{cases}$$

<u>Individual-Specific Random Effects</u>

To mimic real-world data where individual participant's response varies, we modeled random effects for each simulated participant from the population trajectory:

$$\mu_i \; t \; = \mu \; t \; + \alpha_i + \beta_i \cdot t$$

The random effects were drawn from a bivariate normal distribution:

$$\begin{pmatrix} \alpha_i \\ \beta_i \end{pmatrix} \sim N \left(\begin{pmatrix} 0 \\ 0 \end{pmatrix}, \mathbf{\Sigma} \right)$$

with covariance matrix:

$$\mathbf{\Sigma} = \begin{pmatrix} \sigma_\alpha^2 & \rho\sigma_\alpha\sigma_\beta \\ \rho\sigma_\alpha\sigma_\beta & \sigma_\beta^2 \end{pmatrix}$$

where α_i is individual i's deviation from the population mean at baseline, β_i is individual i's deviation from the population slope over 48 hours, σ_α is the between-subject standard deviation in pain scores at baseline, σ_β is the between-subject standard deviation in slope over 48 hours, and ρ is the correlation between the deviations in baseline pain and the slope.

These random effects are generated once per individual and their individual mean trajectory can be computed for the entire study:

$$\mu_i(t_j) = \mu_{\text{base}}(t_j) + \mu_{\text{dose}}(t_j) + \alpha_i + \beta_i \cdot t_j$$

<u>Within-individual temporal correlation and residual error</u>

To account for the correlation of pain scores over time, we modeled the error term using a first-order autoregressive [AR(1)] process:

$$\epsilon_{i,j} = \phi \times \epsilon_{i,j-1} + \eta_{i,j}$$

where ϕ is the AR(1) correlation coefficient, $\epsilon_{i,j}$ is the residual error for individual i at timepoint j, and $\eta_{i,j}$ is the independent residual variability. Note that the AR(1) process indices a correlation between measurements that decreases with distance between time points such that $\mathrm{Corr}(\epsilon_{i,j}, \epsilon_{i,k}) = \phi^{|j-k|}$. This was consistent with the strong observed correlation at lag-1 (adjacent pain measurements) and rapid decay in correlation after lag-1 observed across historical trials.

The AR(1) errors are generated sequentially to create temporal correlation with a consistent residual error term. The first error term is drawn from the marginal distribution:

$$\epsilon_{i,1} \sim N(0, \sigma_\epsilon^2)$$

For subsequent timepoints $(j = 2, 3, \ldots, J)$, we first draw an independent residual variability term:

$$\eta_{i,j} \sim N(0, \sigma_\eta^2), \text{ where } \sigma_\eta = \sigma_\epsilon \times \sqrt{1 - \phi^2}$$

Then, the correlated error is computed by combining the product of the AR(1) correlation and the prior error term with the new residual variability term:

$$\epsilon_{i,j} = \phi \times \epsilon_{i,j-1} + \eta_{i,j}$$

This sequential process creates a series of correlated errors where each error has the desired marginal standard deviation such that $Var(\epsilon_{i,j}) = \sigma_\epsilon^2$ and the desired correlation structure within individual over time.

<u>Computing Final Counterfactual Values</u>

From here, the counterfactual pain scores can be computed as:

$$Y_{i,j}^* = \mu_i(t_j) + \epsilon_{i,j}$$

where we apply bounds to the NRS 0-10 scale and round all numbers to integers:

$$Y_{i,j} = \mathrm{round}(\max(0, \min(Y_{i,j}^*, 10)))$$

If the baseline pain score $(Y_{i,1})$ was less than the inclusion criteria for the simulated trials (i.e., 5 on the NRS), the entire series of pain scores is shifted up by the difference between the minimum allowable baseline value and $Y_{i,1}$, and the logical bounds of 0-10 are re-applied. This process was

to ensure that all simulated individuals meet the minimum baseline pain inclusion criteria, as would be required in an actual clinical trial, while preserving their individual-specific trajectory.

Final Counterfactual Simulation Parameters

The final counterfactual simulation parameters are shown in **Supplemental Table 12**. The base population mean trajectories were based on observed placebo trajectories. Modifications to the mean trajectories and dose effects for the active arms were scaled to produce small, medium, and large treatment effects for an oral analgesic. Variability parameters were set to values somewhat less than observed in prior trials to reflect the lower variability expected without rescue medication. Correlation parameters were based on typical values from the historical trials.

Supplemental Table 12. Simulation parameters by treatment group

Parameter	Placebo	Low Dose	Medium Dose	High Dose
Base population mean trajectory				
Baseline mean	7.0	7.0	7.0	7.0
48-hour mean	4.0	3.5	3.0	2.5
Decay rate (hour^{-1})	0.08	0.08	0.10	0.12
Dose effects on population mean				
Dose 1 drop (δ_1)	0.5	1.0	1.5	2.0
Dose 2 drop (δ_2)	0.25	0.5	0.75	1.0
Rebound factor (r)	1.0	1.0	1.0	1.0
Between-individual variability				
Baseline standard deviation (σ_α)	1.2	1.2	1.2	1.2
Slope standard deviation (σ_β)	0.04	0.04	0.04	0.04
Baseline-slope correlation (ρ)	-0.3	-0.3	-0.3	-0.3
Within-individual temporal correlation				
AR(1) correlation (ϕ)	0.6	0.6	0.6	0.6
Residual standard deviation (σ_ϵ)	1.4	1.4	1.4	1.4

Note: Between-individual and within-individual variability parameters were set modestly lower than observed values from historical trials to reflect the expectation that counterfactual no-rescue trajectories would exhibit less heterogeneity than observed data where rescue use introduces additional variability. The rebound factor of 1.0 indicates complete return to the base exponential recovery trajectory before the next scheduled dose.

Parameter Estimation to Simulate Rescue-Taking Behavior

With counterfactual pain scores generated, the next step is to simulate realistic rescue-taking behavior. For each of the 3 historical bunionectomy trials, we evaluated the probability of taking rescue within 15 minutes of all possible values of NRS from 0 to 10 within each study. (For the

AP-ADF-105 study, VAS scores were converted to NRS by rounding the VAS score divided by 10.) As expected, the probability of taking rescue within 15 minutes was very low at lower pain intensity scores and increased with higher NRS values (**Supplemental Figure 13**, bottom panel). There were no apparent differences in the probability of taking pain intensity scores at different NRS scores by treatment group, although the placebo groups in each study tended to have the highest prevalence of severe pain scores (**Supplemental Figure 13**, upper panel). There was no evidence that the probability of taking rescue at particular pain intensity scores varied by time, so probabilities were averaged across trials for the values used in the simulation (**Supplemental Table 13**).

Supplemental Figure 13. Distribution of NRS scores and probability of using rescue medication within 15 minutes of scheduled pain assessment at each NRS value by trial

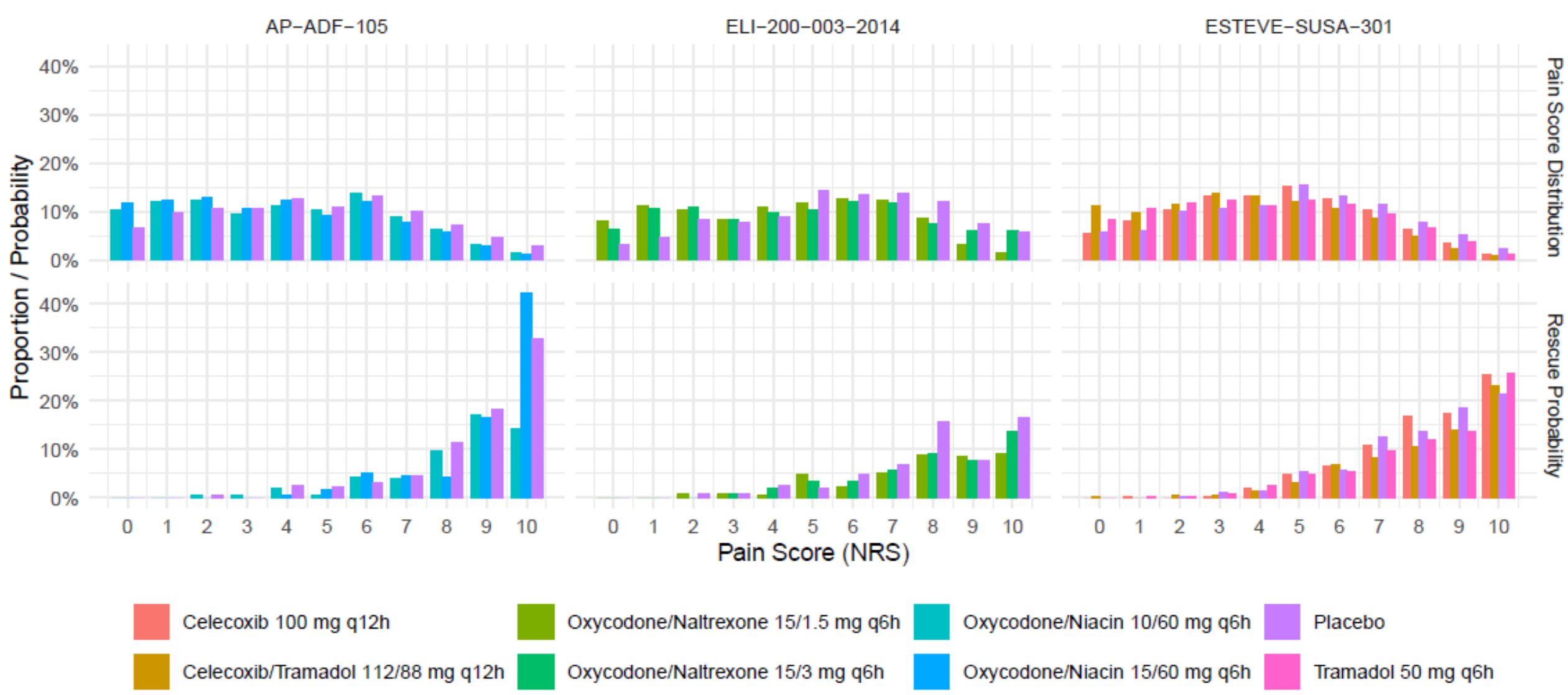

Supplemental Table 13. Rescue probabilities used in simulation

NRS value	Probability of taking rescue within 15 minutes
0	0.000416
1	0.000708
2	0.00304
3	0.00578
4	0.0177
5	0.0381
6	0.0534
7	0.084
8	0.122
9	0.144
10	0.221

Details on Rescue Effect Simulation

With a simulated counterfactual dataset, rescue use was simulated sequentially on a participant-by-participant basis. After an initial 90-minute lock-out period consistent with clinical trial practice, simulated participants had the opportunity to take rescue medication at each 15-minute timepoint based on their current counterfactual pain score with empirical probabilities derived from historical trials (**Supplemental Table 13**). Once rescue medication was taken, they entered a 6-hour lockout period (or rescue window) during which no additional rescue could be taken. Rescue effects $\Delta_{i,j}$ were modeled using a similar approach to the generation of counterfactuals with 3 components: the population-level effect, between-individual heterogeneity in rescue response, and within-individual temporal correlation and residual variation.

First, we consider the population-level effect of rescue over time, which is derived from the reference distribution. For timepoint j where rescue medication is active, we let $s_j = t_j - t^*_{i,\text{last}}$ be the time since the last rescue administration in hours, $\gamma_{\text{ref}}(s)$ be the reference-based population mean rescue effect at time s after rescue dosing and $\sigma_{\text{ref}}(s)$ be the reference-based population standard deviation at time s after rescue dosing.

The between-individual heterogeneity was modeled using random intercepts, which remained constant across all rescue episodes. This allowed each individual to have a similar response to rescue medication across all rescue episodes. For each individual i, we draw a standard normal deviate:

$$\zeta_i \sim N(0,1)$$

which is scaled by the between-subject standard deviation to produce the individual-specific effect:

$$\xi_i \; s \;\; = \zeta_i \times \sigma_{\text{between}}(s)$$

The between-subject standard deviation is set at each post-rescue timepoint s as:

$$\sigma_{\text{between}} \; s \;\; = \sigma_{\text{ref}}(s) \times \sqrt{\rho_{\text{between}}}$$

where ρ_{between} is the proportion of variance in rescue variability attributable to between-individual differences.

The within-individual residual variability follows an AR(1) process identical in structure to the counterfactual pain model. For each rescue episode, residuals are generated as:

$$\epsilon_{i,j}(s) = \phi \times \epsilon_{i,j-1}(s) + \eta_{i,j}(s)$$

where the marginal variance $\text{Var}(\epsilon_{i,j}(s)) = \sigma^2_{\text{within}}(s)$, and

$$\sigma_{\text{within}} \; s \;\; = \sqrt{1 - \rho_{\text{between}}} \times \sigma_{\text{ref}}(s)$$

The same sequential process to generate errors as was used for counterfactual pain scores was used such that, at the start of each new rescue episode, the first time point is drawn from the marginal distribution:

$$\epsilon_{i,1} \; s \;\; \sim N(0, \sigma^2_{\text{within}} \; s \;)$$

And for subsequent timepoints $j \geq 2$ that fall in the rescue window, we first draw an independent residual variability term:

$\eta_{i,j}(s_j) \sim N(0, \sigma^2_\eta(s_j))$, where $\sigma_\eta(s_j) = \sigma_{\text{within}}(s_j) \times \sqrt{1 - \phi^2}$

Then, the correlated error is computed by combining the product of the AR(1) correlation and the prior error term with the new residual variability term:

$$\epsilon_{i,j}(s_j) = \phi \times \epsilon_{i,j-1}(s_{j-1}) + \eta_{i,j}(s_j)$$

Thus, for an individual i at timepoint j when rescue is active:

$$\Delta_{i,j} = \gamma(s_j) + \zeta_i \times \sigma_{\text{between}}(s_j) + \epsilon_{i,j}(s_j)$$

where s_j reflects the time (in hours) since the last rescue administration. It naturally follows that when rescue is not active, $\Delta_{i,j} = 0$.

Computing Observed Values after Counterfactual and Rescue Simulations

Previously, we derived final counterfactual pain scores under a no-rescue scenario, the target of inference for the hypothetical estimand, using historical data from the placebo arms of prior bunionectomy trials. We also simulated realistic effects of rescue based on the anticipated effect of the rescue drug and implemented expected rescue-taking behavior based on prior bunionectomy trials. Thus, to simulate the observed pain scores under the assumption of additivity, we incorporate the rescue effect $(\Delta_{i,j})$ onto the counterfactual pain scores $(Y_{i,j})$:

$$Y_{i,j}^{*,\text{obs}} = Y_{i,j} + \Delta_{i,j}$$

where, as before, we apply bounds to the NRS 0-10 scale and ensure all pain scores are integers:

$$Y_{i,j}^{\text{obs}} = \text{round}(\max(0, \min(Y_{i,j}^{*,\text{obs}}, 10)))$$

Evaluating Robustness to Misspecification

To evaluate the robustness of MIRA to misspecification of the true rescue effect distribution, we simulated three different scenarios for the effect of rescue by applying adjustment factors to the simulated rescue effect. The adjusted rescue effect is simply:

$$\Delta_{i,j}^{(\kappa)} = \kappa \times \Delta_{i,j}$$

which is applied to the counterfactual pain score to produce an adjusted observed pain score. The three scenarios we evaluated were:

- $\kappa = 1.0$, where the simulated rescue effects exactly match the reference distribution. This represents a situation where the reference distribution of rescue effects for MIRA are correctly specified.
- $\kappa = 1.5$, where the simulated rescue effects are 50% stronger than the reference distribution. This represents a situation where the reference distribution of rescue effects for MIRA are misspecified. In this case, MIRA will systematically underestimate the true effect of rescue on pain scores.
- $\kappa = 0.5$, where the simulated rescue effects are 50% weaker than the reference distribution. This also represents a situation where the reference distribution of rescue effects for MIRA are misspecified. In this case, MIRA will systematically overestimate the true effect of rescue on pain scores.

Details on SPID Calculations

The primary estimand was the hypothetical estimand for $SPID_{0\text{-}48}$ calculated using the trapezoidal method. For each individual i, SPID was calculated as:

$$\mathrm{SPID}_i = \sum_{j=1}^{J-1} \frac{PID_{i,j} + PID_{i,j+1}}{2} (t_{i,j+1} - t_{i,j})$$

where J is the total number of scheduled and unscheduled pain assessments for the participant during the time period of interest, $PID_{i,j}$ is the pain intensity difference from baseline at assessment j $(Y_{i,1} - Y_{i,j})$, and $t_{i,j+1} - t_{i,j}$ is the time between measurements. Rescue medication was the only intercurrent event that needed to be addressed in the simulations since we simulated the data without intermittent missing data or early dropout. Therefore, J reflects the total number

of scheduled pain assessments plus the number of times the individual took rescue medication, and the Jth pain assessment is at 48 hours.

Despite simulating counterfactual pain scores every 15 minutes for the purposes of both generating scheduled timepoints and facilitating the simulation of rescue-taking behavior, only the pain scores taken at scheduled timepoints and at the taking of rescue medication were incorporated into SPID calculations.

Details on Multiple Imputation for Rescue Adjustment (MIRA)

MIRA is a reference-based multiple imputation procedure that estimates counterfactual pain scores by removing the estimate effect of rescue from observed pain scores. For timepoints affected by rescue medication, the counterfactual pain score is imputed as:

$$Y_{i,j} = Y_{i,j}^{obs} - \widetilde{\Delta}_{i,j}$$

where $\widetilde{\Delta}_{i,j}$ is a sampled effect of the rescue medication from the reference distribution. This imputation is repeated M times, creating M complete datasets. Treatment effects can then be estimated in each imputed dataset and pooled using Rubin's rules or alternate approach.

More specifically, for each imputation $m = 1, \dots, M$, for each rescue-affected timepoint j where $s_j = t_j - t_{i,\text{last}}^*$ as previously defined, the time since last rescue medication use, we sample the rescue effect as:

$$\widetilde{\Delta}_{i,j}^{(m)} \sim N(\gamma(s_j), \sigma_{\text{ref}}^2(s_j))$$

where $\gamma(s_j)$ is the estimated mean treatment effect and $\sigma_{\text{ref}}^2(s_j)$ is the estimate variance of the treatment effect of the rescue medication from the reference distribution at time s_j. The imputed counterfactual is computed as:

$$Y_{i,j}^{(m)} = \max\ (0, \min\ (10, Y_{i,j}^{obs} - \widetilde{\Delta}_{i,j}^{\ m}\ )$$

to respect the bounds of the 0-10 NRS scale.

Thus, the mth imputed dataset contains:

$$Y_{i,j}^{(m)} = \begin{cases} Y_{i,j}^{\ m} & \text{if rescue active} \\ Y_{i,j}^{obs} & \text{otherwise} \end{cases}$$

from which data can be analyzed and results pooled using established approaches for multiple imputation (e.g., Rubin's rules).

All code to perform the simulation studies reported in the primary manuscript may be found on GitHub: https://github.com/cjm2718/MIRA.

Supplemental Tables and Figures of Results

Supplemental Table 14. Bias for group-level estimates

Treatment Group	Analysis Method	Rescue Effect	Mean Bias	SD Bias	Mean % Bias
Placebo	SPID-Observed	Expected	17.16	2.592	16.0%
Placebo	SPID-Observed	0.5x Expected	7.81	1.400	7.3%
Placebo	SPID-Observed	1.5x Expected	23.75	3.429	22.2%
Placebo	SPID-LOCF	Not Dependent	-21.25	1.348	-19.8%
Placebo	SPID-BOCF	Not Dependent	-43.54	2.615	-40.6%
Placebo	SPID-WOCF	Not Dependent	-82.87	2.721	-77.4%
Placebo	SPID-MIRA	Expected	-3.04	2.232	-2.8%
Placebo	SPID-MIRA	0.5x Expected	-11.93	1.267	-11.1%
Placebo	SPID-MIRA	1.5x Expected	3.38	2.909	3.2%
Low	SPID-Observed	Expected	14.65	2.368	11.6%
Low	SPID-Observed	0.5x Expected	6.73	1.292	5.3%
Low	SPID-Observed	1.5x Expected	19.87	3.115	15.7%
Low	SPID-LOCF	Not Dependent	-20.13	1.287	-15.9%
Low	SPID-BOCF	Not Dependent	-46.33	2.482	-36.5%
Low	SPID-WOCF	Not Dependent	-77.05	2.766	-60.8%
Low	SPID-MIRA	Expected	-4.03	2.054	-3.2%
Low	SPID-MIRA	0.5x Expected	-11.53	1.194	-9.1%
Low	SPID-MIRA	1.5x Expected	1.05	2.649	0.8%
Medium	SPID-Observed	Expected	11.06	1.989	7.1%
Medium	SPID-Observed	0.5x Expected	5.18	1.101	3.3%
Medium	SPID-Observed	1.5x Expected	14.50	2.593	9.4%
Medium	SPID-LOCF	Not Dependent	-18.23	1.209	-11.7%
Medium	SPID-BOCF	Not Dependent	-47.48	2.486	-30.6%
Medium	SPID-WOCF	Not Dependent	-67.79	2.830	-43.7%
Medium	SPID-MIRA	Expected	-5.03	1.714	-3.2%
Medium	SPID-MIRA	0.5x Expected	-10.54	1.037	-6.8%
Medium	SPID-MIRA	1.5x Expected	-1.70	2.190	-1.1%
High	SPID-Observed	Expected	7.90	1.652	4.3%
High	SPID-Observed	0.5x Expected	3.78	0.927	2.1%
High	SPID-Observed	1.5x Expected	9.97	2.151	5.5%
High	SPID-LOCF	Not Dependent	-15.80	1.133	-8.7%
High	SPID-BOCF	Not Dependent	-45.02	2.460	-24.7%
High	SPID-WOCF	Not Dependent	-57.69	2.842	-31.7%
High	SPID-MIRA	Expected	-5.43	1.434	-3.0%
High	SPID-MIRA	0.5x Expected	-9.23	0.909	-5.1%
High	SPID-MIRA	1.5x Expected	-3.45	1.822	-1.9%

Since absolute and percent bias are not affected by sample size, only results for trials with N=200 per group are shown.

Supplemental Table 15. Bias for treatment effects

Contrast	Analysis Method	Rescue Effect	Mean Bias	SD Bias	Mean % Bias
Small	SPID-Observed	Expected	-2.51	3.51	-12.5%
Small	SPID-Observed	0.5x Expected	-1.08	1.90	-5.3%
Small	SPID-Observed	1.5x Expected	-3.89	4.64	-19.4%
Small	SPID-LOCF	Not Dependent	1.12	1.85	6.4%
Small	SPID-BOCF	Not Dependent	-2.79	2.70	-13.9%
Small	SPID-WOCF	Not Dependent	5.81	3.77	31.1%
Small	SPID-MIRA	Expected	-0.99	3.03	-4.6%
Small	SPID-MIRA	0.5x Expected	0.41	1.73	2.4%
Small	SPID-MIRA	1.5x Expected	-2.32	3.93	-11.4%
Medium	SPID-Observed	Expected	-6.15	3.27	-12.8%
Medium	SPID-Observed	0.5x Expected	-2.65	1.78	-5.5%
Medium	SPID-Observed	1.5x Expected	-9.34	4.29	-19.4%
Medium	SPID-LOCF	Not Dependent	3.01	1.82	6.3%
Medium	SPID-BOCF	Not Dependent	-3.97	2.78	-8.2%
Medium	SPID-WOCF	Not Dependent	15.18	3.76	31.6%
Medium	SPID-MIRA	Expected	-2.04	2.82	-4.2%
Medium	SPID-MIRA	0.5x Expected	1.38	1.64	2.9%
Medium	SPID-MIRA	1.5x Expected	-5.14	3.64	-10.7%
Large	SPID-Observed	Expected	-9.22	3.07	-12.3%
Large	SPID-Observed	0.5x Expected	-4.00	1.68	-5.3%
Large	SPID-Observed	1.5x Expected	-13.74	4.04	-18.3%
Large	SPID-LOCF	Not Dependent	5.43	1.74	7.3%
Large	SPID-BOCF	Not Dependent	-1.56	2.84	-2.1%
Large	SPID-WOCF	Not Dependent	25.14	3.73	33.6%
Large	SPID-MIRA	Expected	-2.37	2.64	-3.2%
Large	SPID-MIRA	0.5x Expected	2.72	1.54	3.6%
Large	SPID-MIRA	1.5x Expected	-6.80	3.42	-9.1%

Since absolute and percent bias is not affected by sample size, only results for trials with N=200 per group are shown.

Supplemental Figure 14. Empirical standard errors for treatment contrasts (Panel A) and group-level estimates (Panel B)

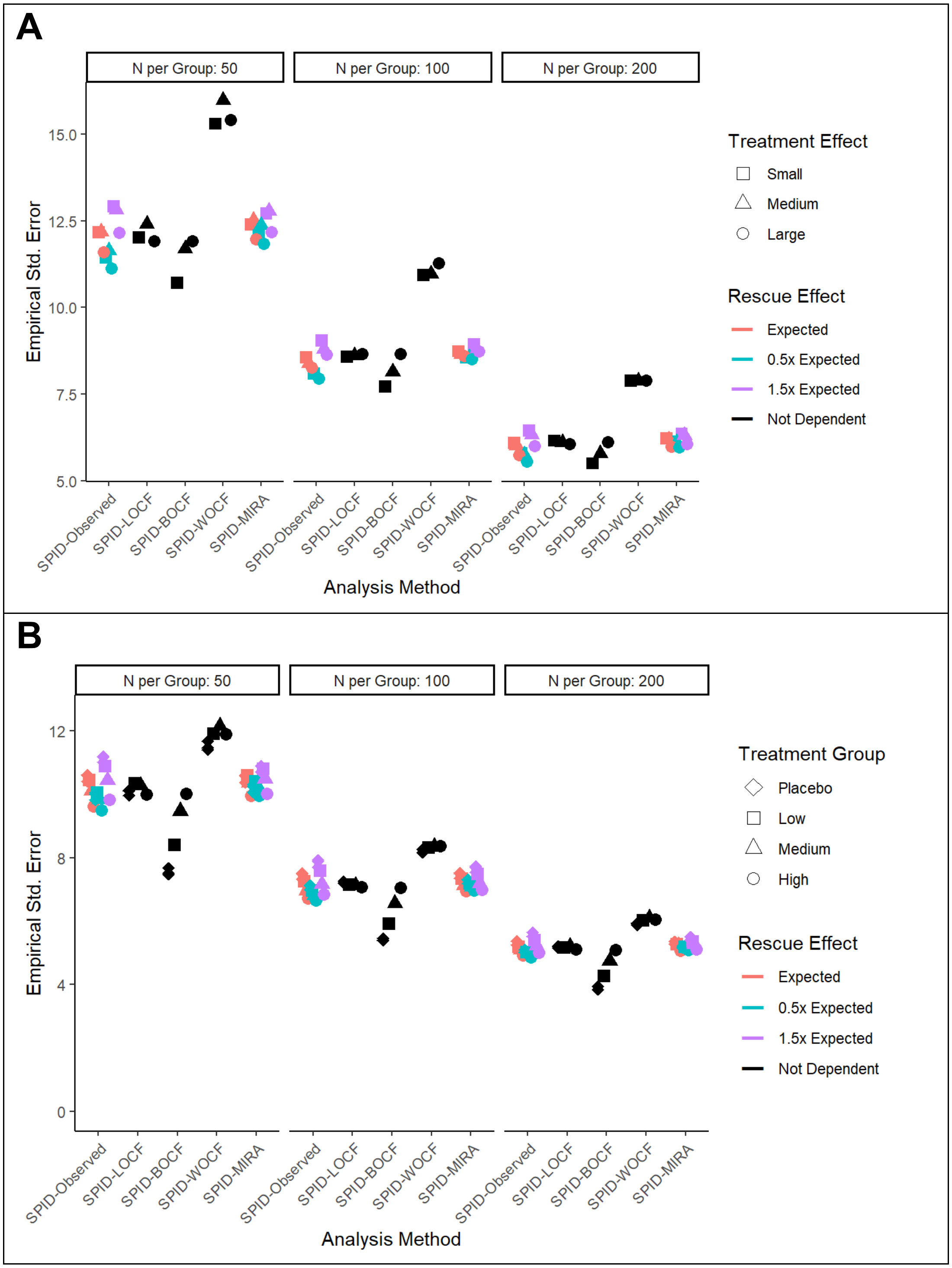

Supplemental Figure 15. Root mean square error for treatment contrasts (Panel A) and group-level estimates (Panel B)

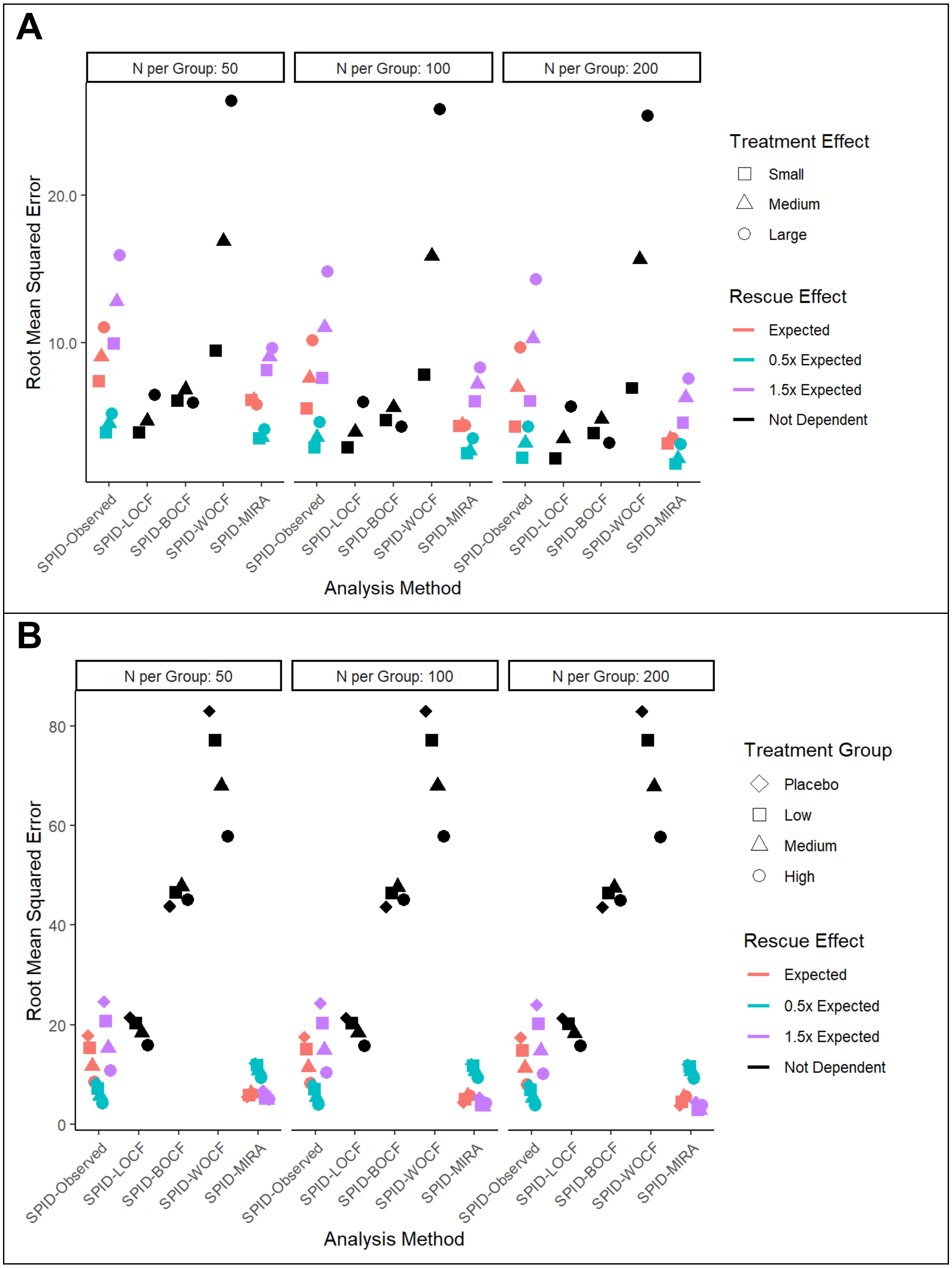

Supplemental Figure 16. Type-I error rates (Panel A) and power (Panel B)

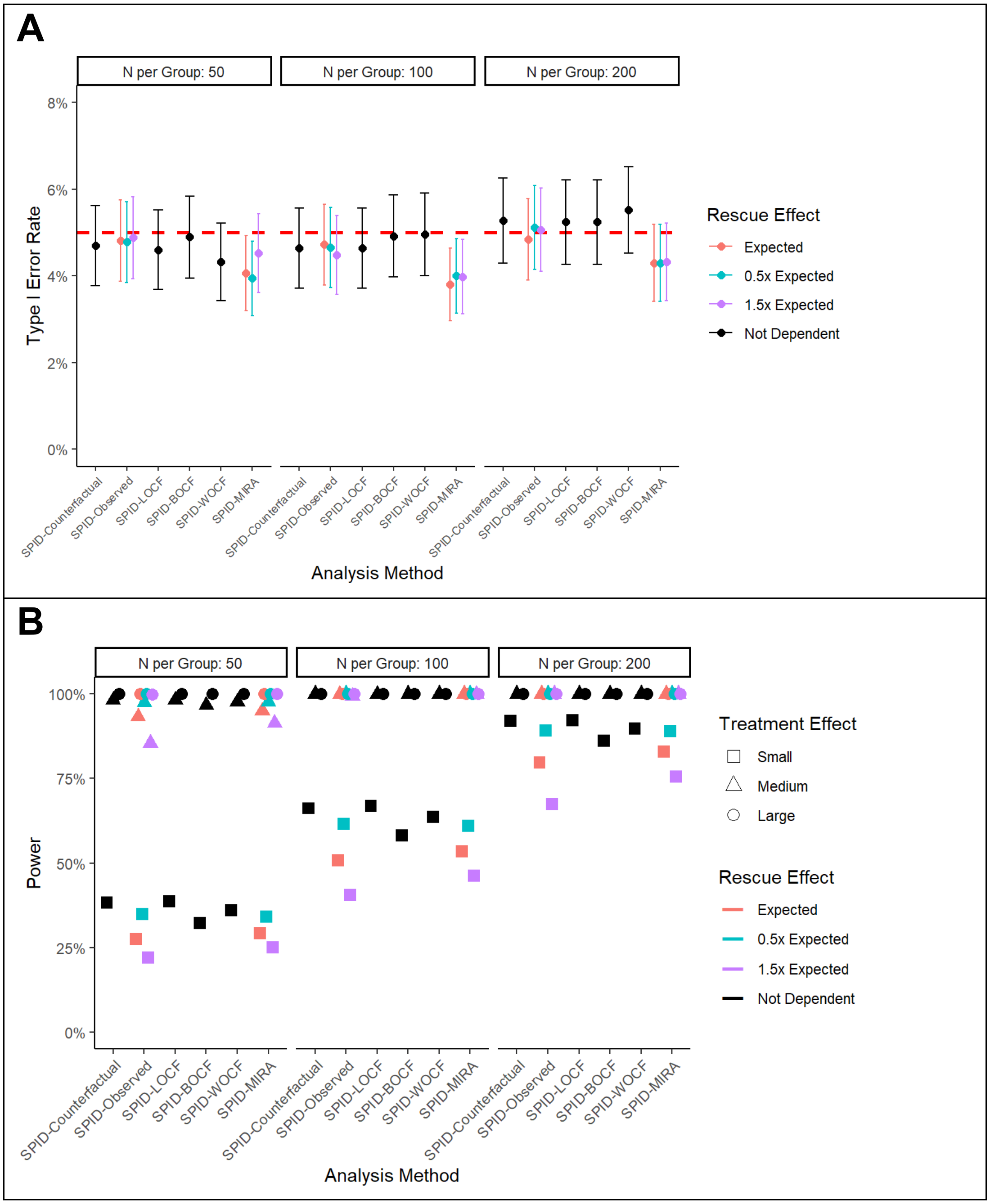

Supplemental Figure 17. Time to first use of HC/APAP rescue medication over first 6 hours in bunionectomy trials DIC3-08-04, IND3-08-04b, and IND3-10-06

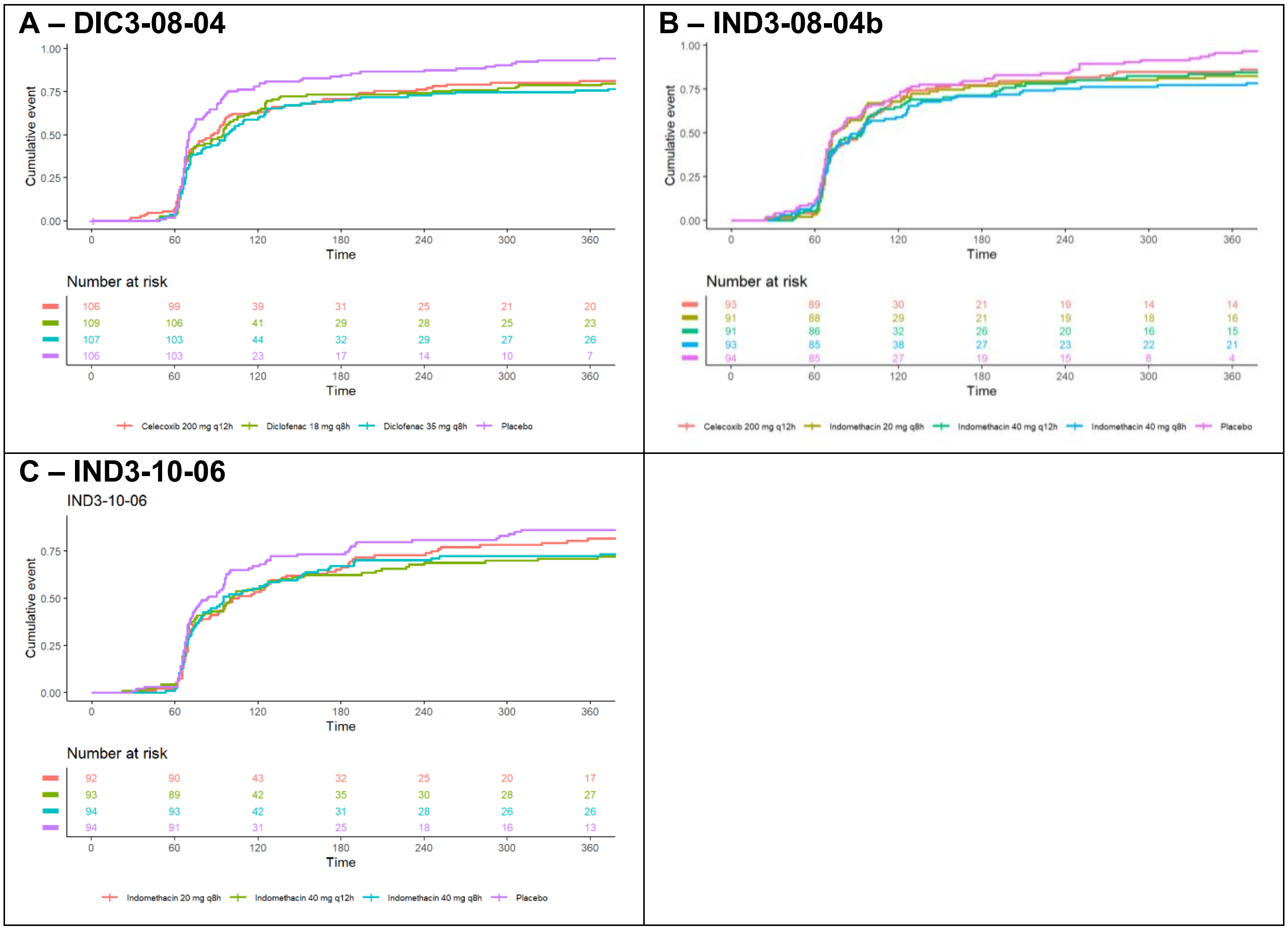

Supplemental Figure 18. Mean pain scores over time for DIC3-08-04 by imputation method for rescue medication and treatment group

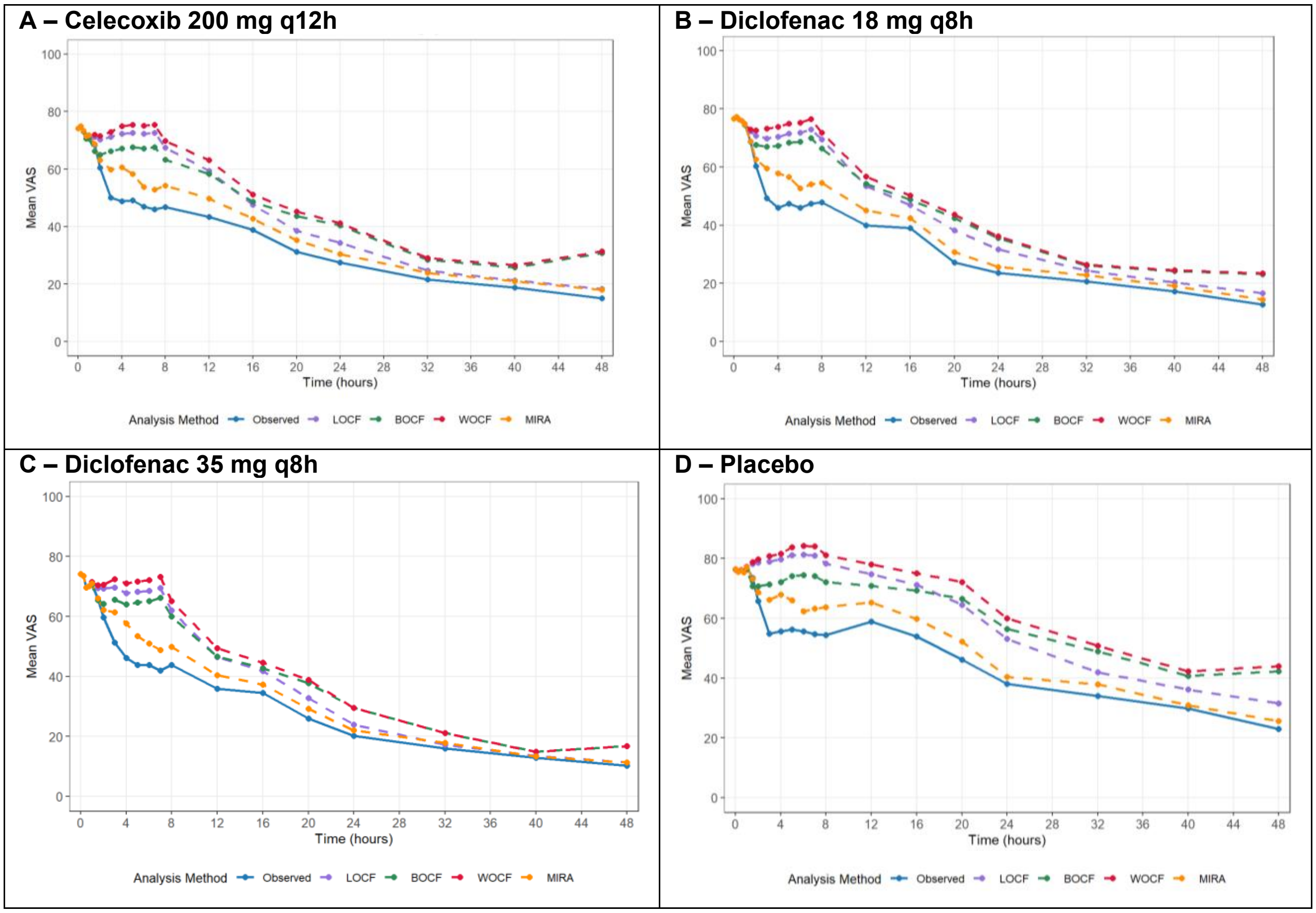

Supplemental Figure 19. Mean pain scores over time for IND3-08-04b by imputation method for rescue medication and treatment group

A – Celecoxib 200 mg q12h

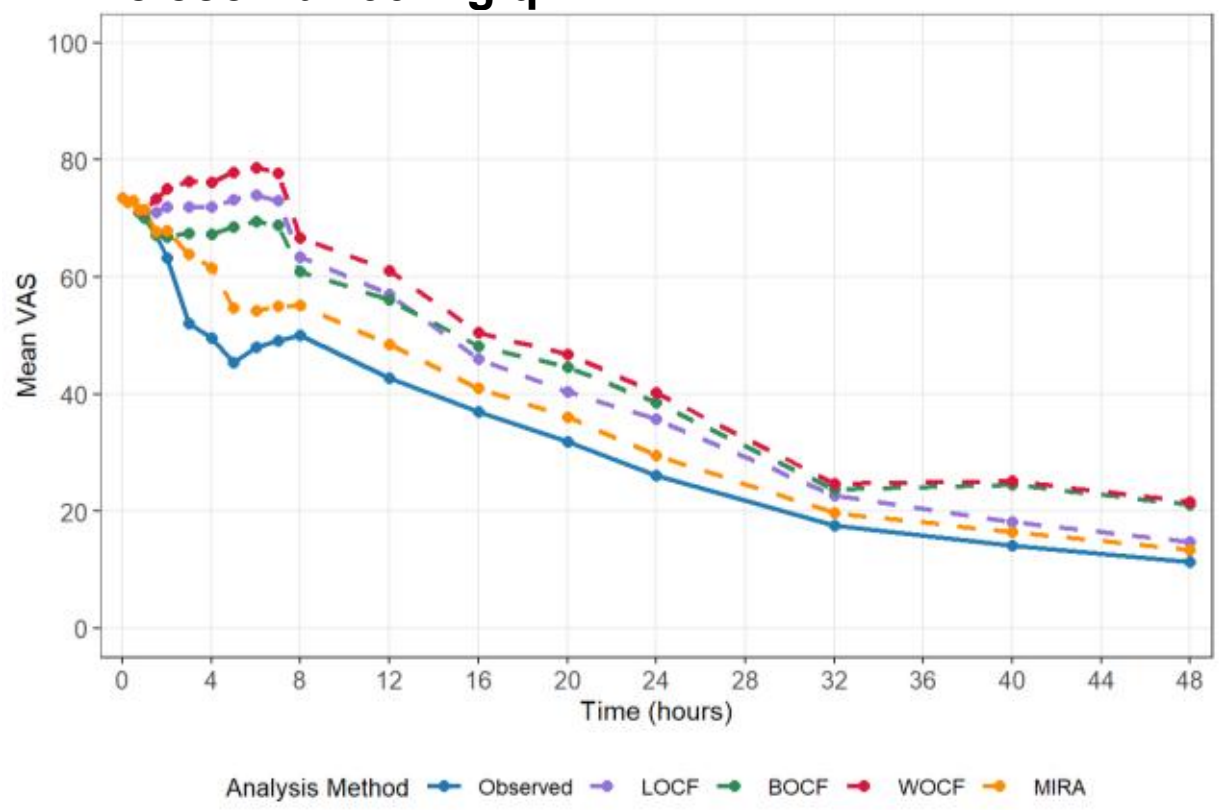

B – Indomethacin 20 mg q8h

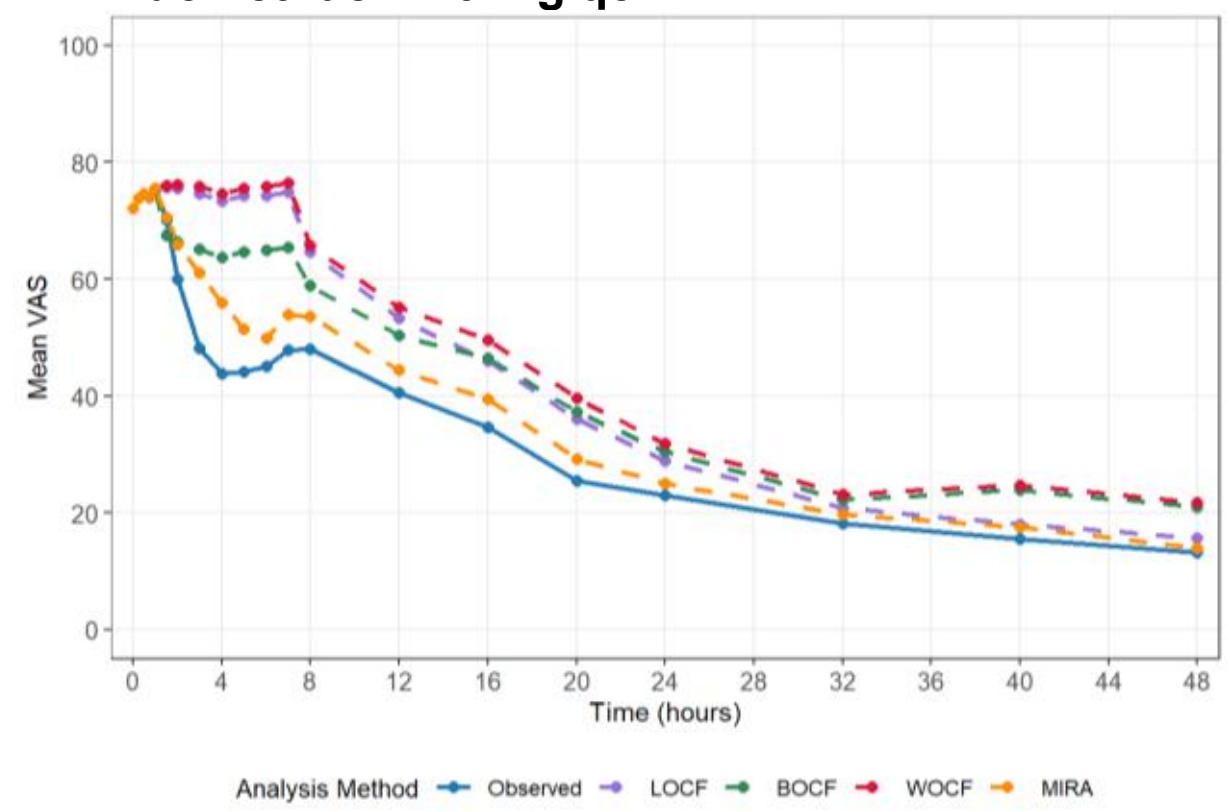

C – Indomethacin 40 mg q12h

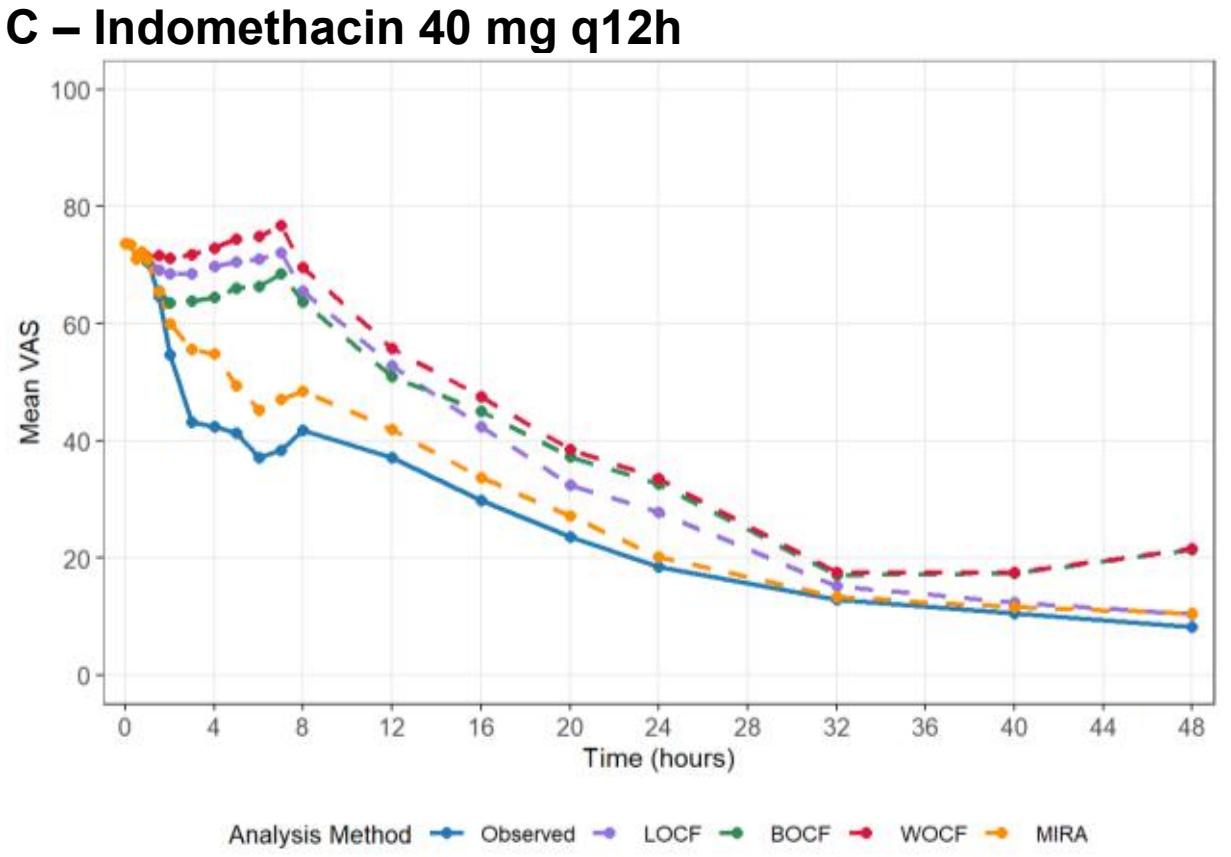

D – Indomethacin 40 mg q8h

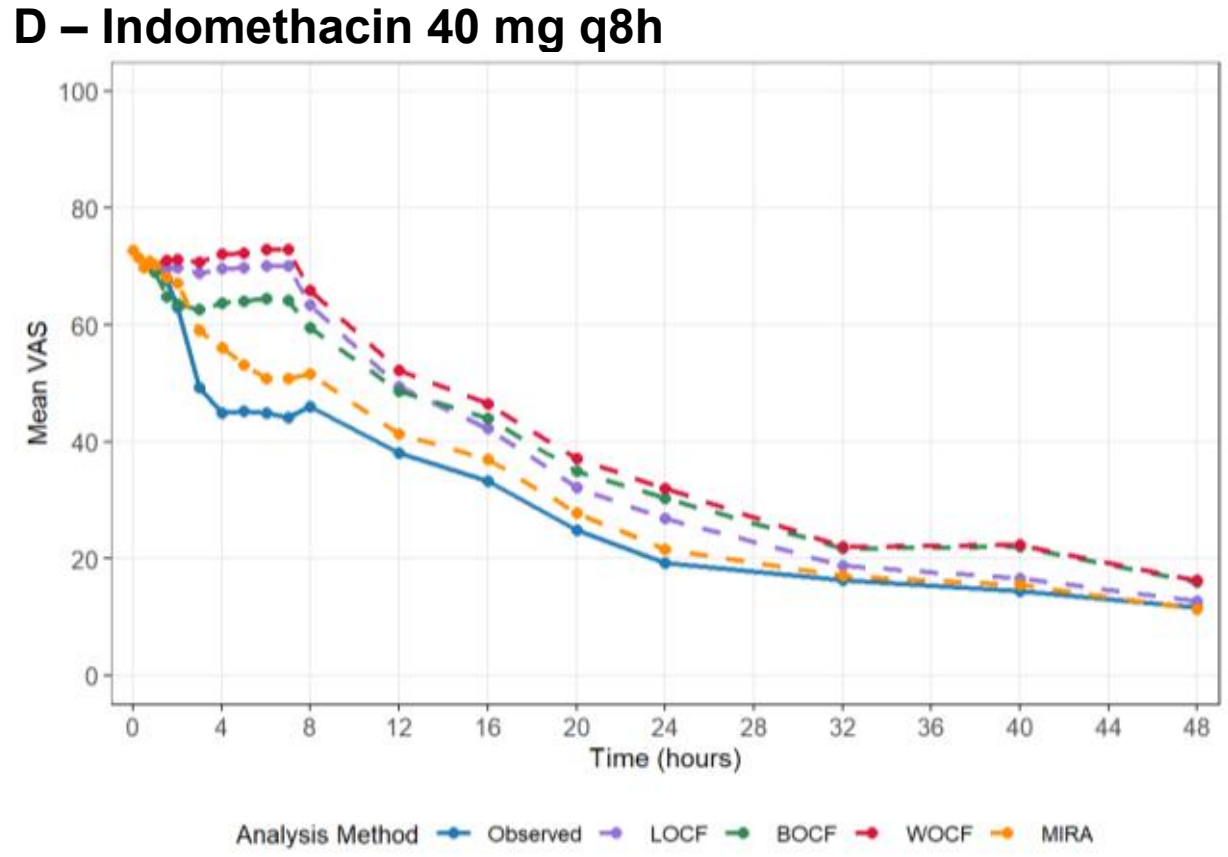

E – Placebo

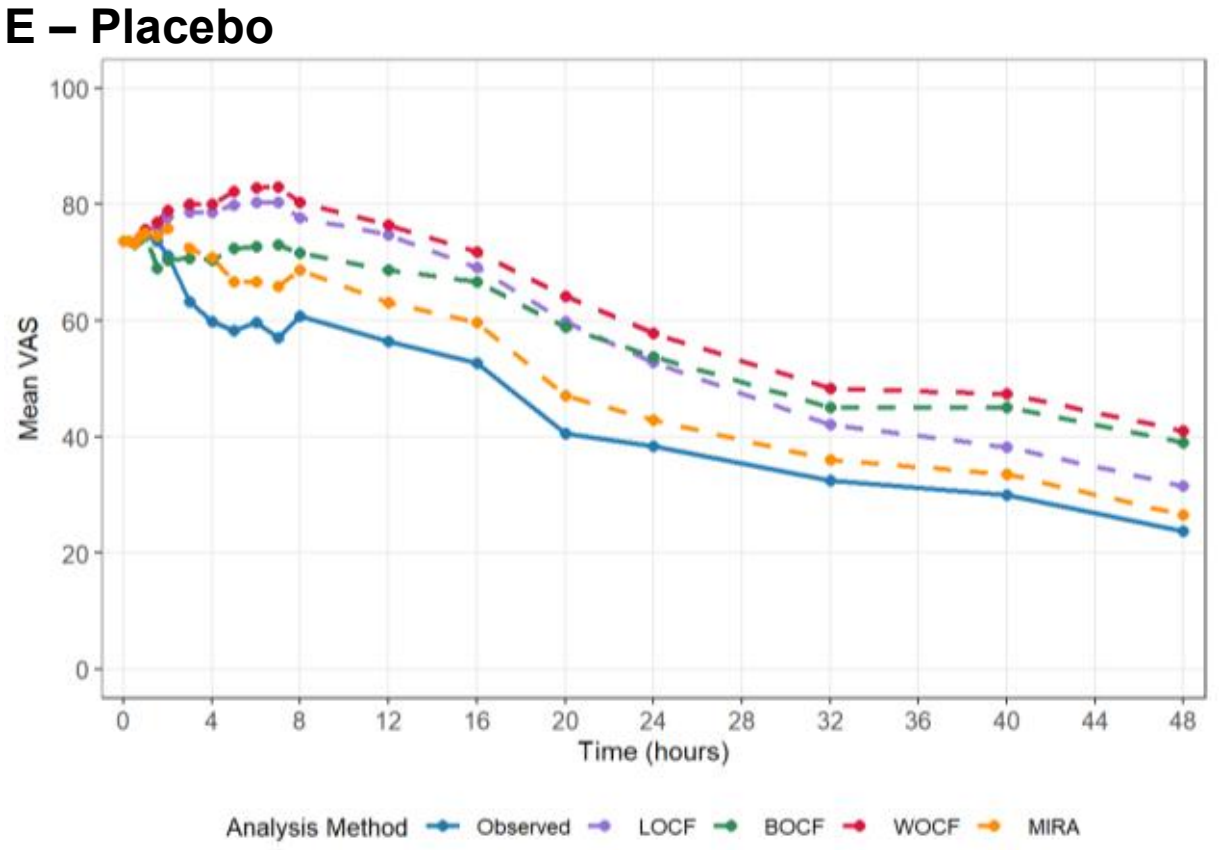

Supplemental Figure 20. Mean pain scores over time for IND3-10-06 by imputation method for rescue medication and treatment group

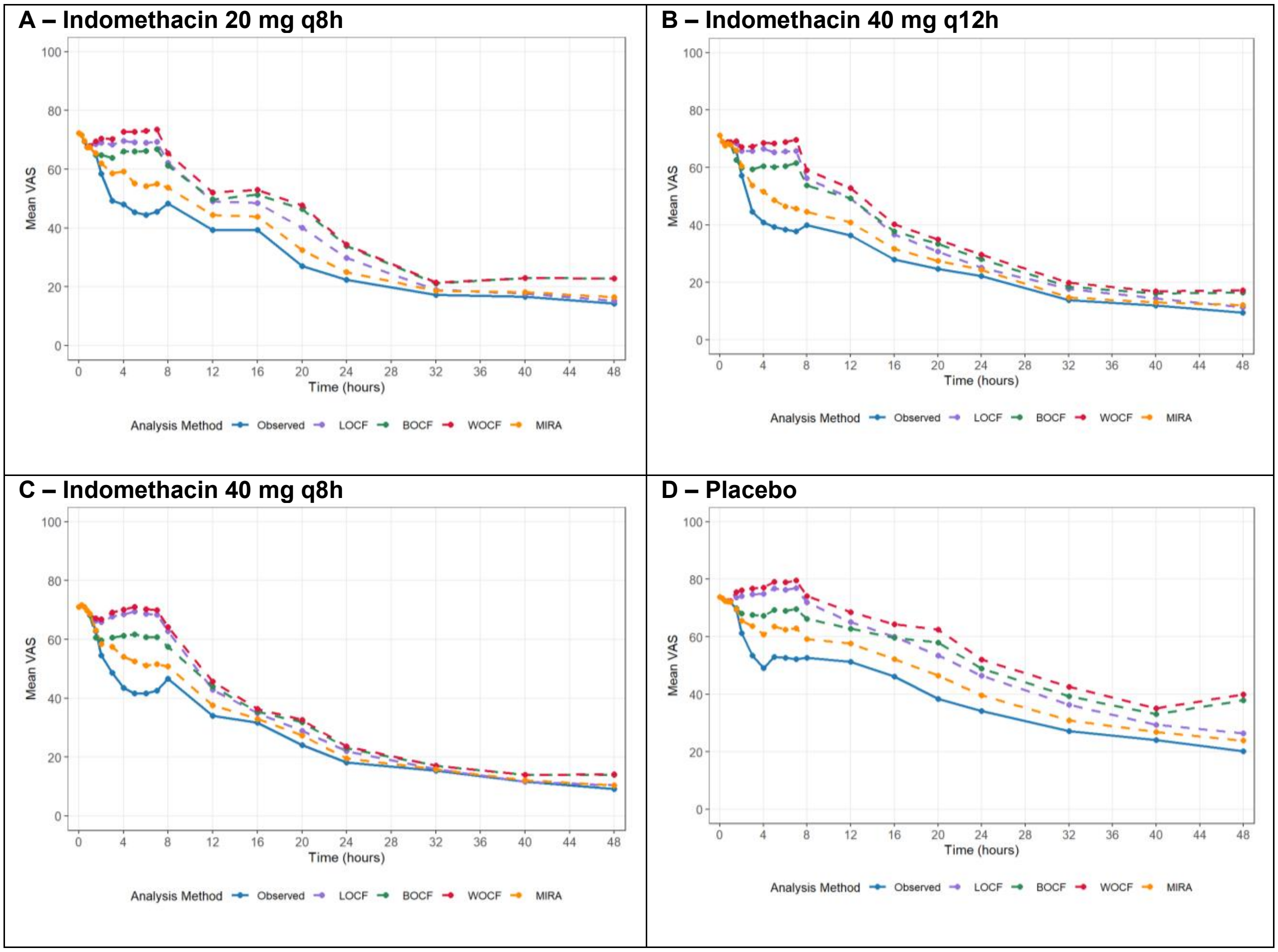

Supplemental Figure 21. Estimated mean treatment differences (95% CI) on SPID for active treatment groups versus placebo by treatment group in bunionectomy trials DIC3-08-04, IND3-08-04b, and IND3-10-06

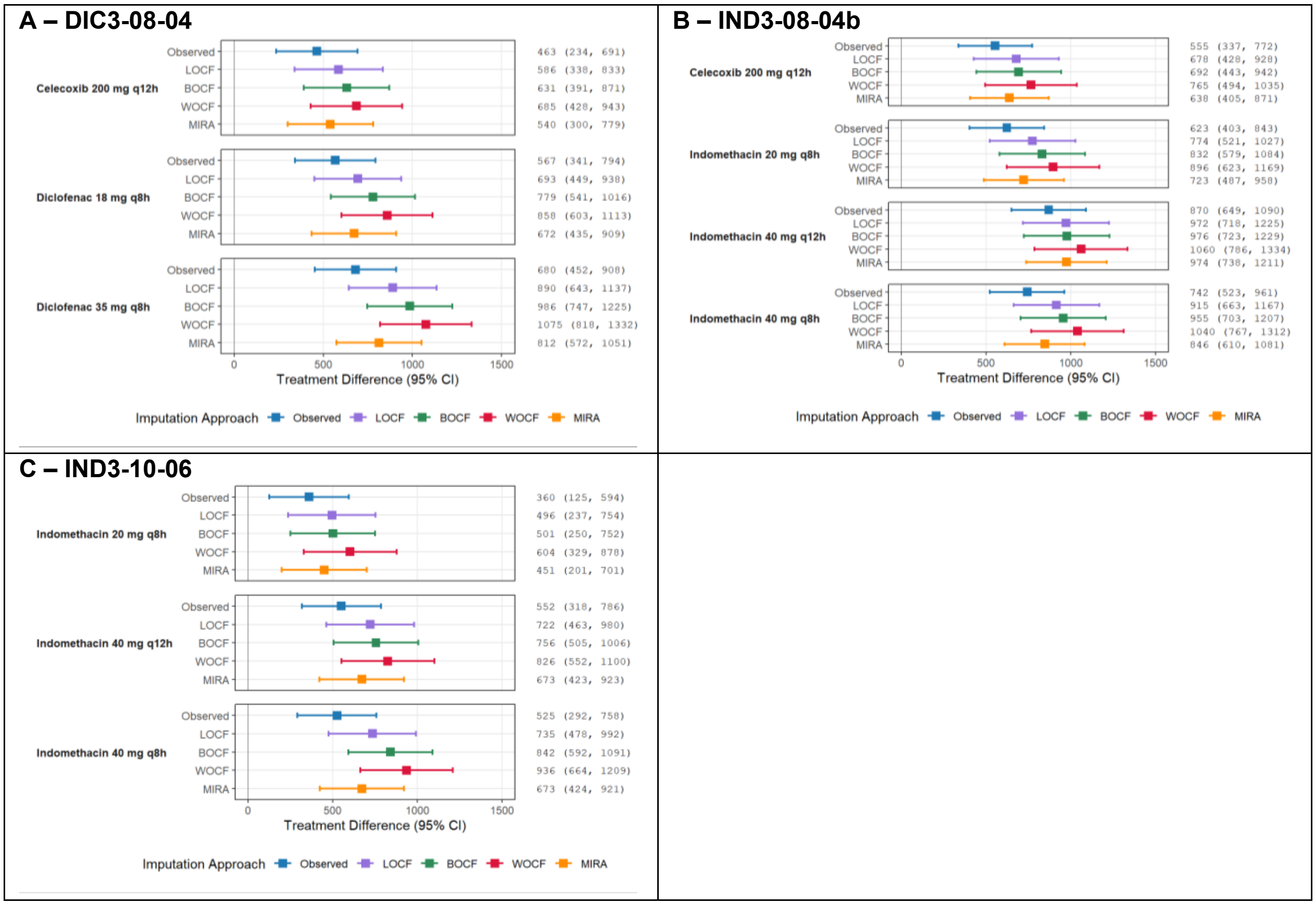

Supplemental Figure 22. Adjusted marginal mean (95% CI) SPID estimates by treatment group in bunionectomy trials DIC3-08-04, IND3-08-04b, and IND3-10-06

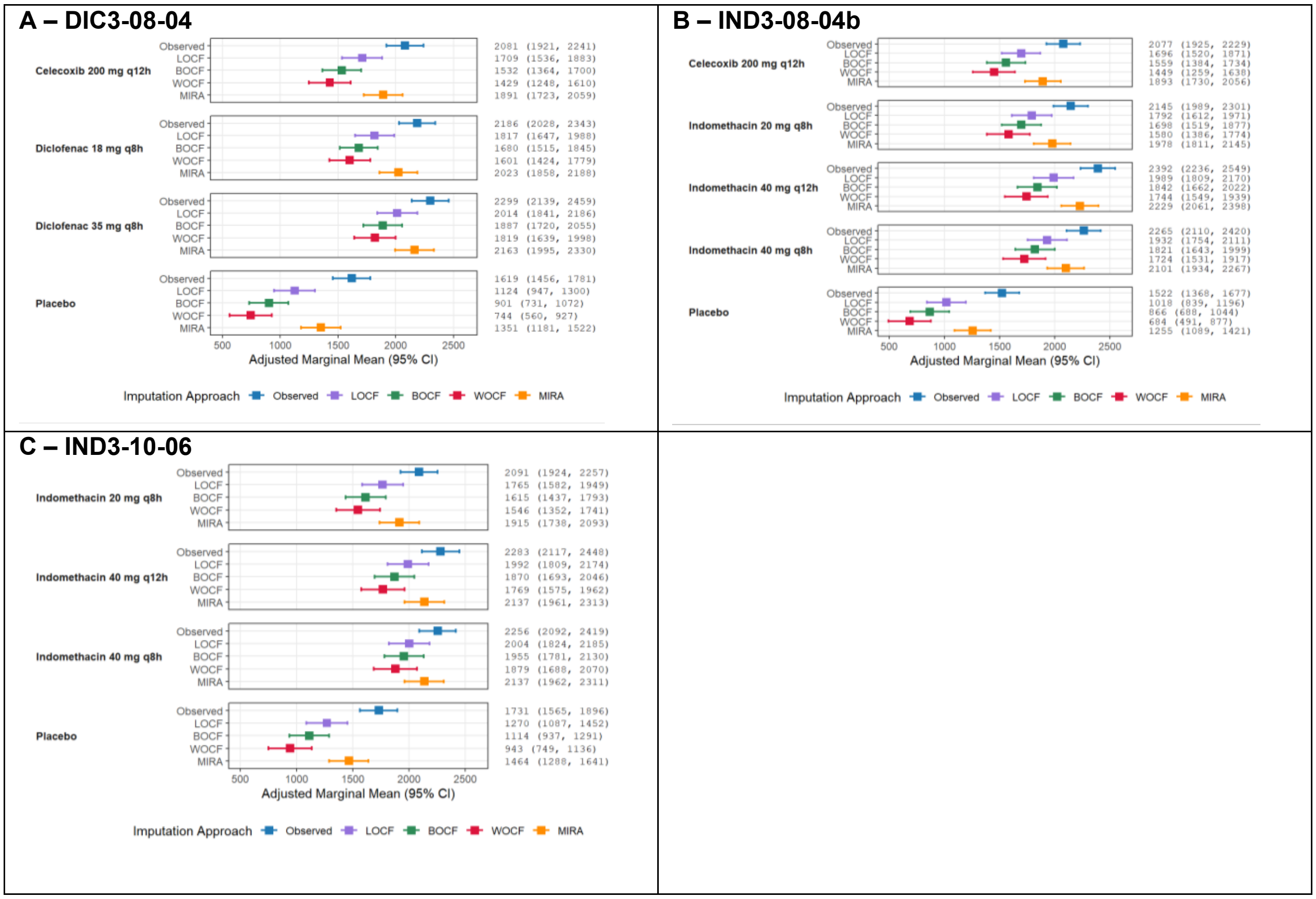

Supplement: Supplementary file 1 [file jop-167-2439-s001.pdf]
